# Fruit load induces changes in global gene expression and in abscisic acid (ABA) and indole acetic acid (IAA) homeostasis in citrus buds

**DOI:** 10.1093/jxb/eru148

**Published:** 2014-04-04

**Authors:** Liron Shalom, Sivan Samuels, Naftali Zur, Lyudmila Shlizerman, Adi Doron-Faigenboim, Eduardo Blumwald, Avi Sadka

**Affiliations:** ^1^Department of Fruit Trees Sciences, Agricultural Research Organization, The Volcani Center, Bet Dagan 50250, Israel; ^2^The Robert H. Smith Faculty of Agriculture, Food and Environment, The Hebrew University of Jerusalem, Rehovot, Israel; ^3^Department of Plant Sciences, University of California, Davis, CA 95616, USA

**Keywords:** Abscisic acid, alternate bearing, auxin, bud, citrus, flowering, fruit load.

## Abstract

Many fruit trees undergo cycles of heavy fruit load (ON-Crop) in one year, followed by low fruit load (OFF-Crop) the following year, a phenomenon known as alternate bearing (AB). The mechanism by which fruit load affects flowering induction during the following year (return bloom) is still unclear. Although not proven, it is commonly accepted that the fruit or an organ which senses fruit presence generates an inhibitory signal that moves into the bud and inhibits apical meristem transition. Indeed, fruit removal from ON-Crop trees (de-fruiting) induces return bloom. Identification of regulatory or metabolic processes modified in the bud in association with altered fruit load might shed light on the nature of the AB signalling process. The bud transcriptome of de-fruited citrus trees was compared with those of ON- and OFF-Crop trees. Fruit removal resulted in relatively rapid changes in global gene expression, including induction of photosynthetic genes and proteins. Altered regulatory mechanisms included abscisic acid (ABA) metabolism and auxin polar transport. Genes of ABA biosynthesis were induced; however, hormone analyses showed that the ABA level was reduced in OFF-Crop buds and in buds shortly following fruit removal. Additionally, genes associated with Ca^2+^-dependent auxin polar transport were remarkably induced in buds of OFF-Crop and de-fruited trees. Hormone analyses showed that auxin levels were reduced in these buds as compared with ON-Crop buds. In view of the auxin transport autoinhibition theory, the possibility that auxin distribution plays a role in determining bud fate is discussed.

## Summary

Fruit removal from heavily loaded citrus trees induces Ca^2+^-dependent auxin polar transport and reduces auxin content in the bud, suggesting hormone involvement in the control of alternate bearing.

## Introduction

Fruit trees exhibit two major multiannual reproductive strategies (Goldschmidt, 2013). In the first, the amount of fruit produced allows a sufficient amount of vegetative growth to support production of an ample number of flowers during the following year (return bloom). Such trees, including fig and some orange and grapefruit cultivars, are defined as regular bearers. They are characterized by a relatively stable multiannual yield, and usually possess efficient mechanism(s) to control excess fruit production. A second strategy is also used by trees that bear a heavy fruit load (ON-Crop) in one year, which inhibits return bloom and vegetative growth the next year (Monselise and Goldschmidt, 1982). Thus, the second year is characterized by low yield (OFF-Crop) and high vegetative growth. Such trees, including olive, pistachio, mandarins, and many others, are defined as alternate or biannual bearers and they are usually characterized by low self-thinning ability (Goldschmidt, 2013). Alternate bearer cultivars present a serious economic problem to fruit growers. Therefore, chemical or manual fruit thinning are common practices in their cultivation (Dennis, 2000). In citrus culture, low temperatures during the autumn and winter are a major factor in inducing flowering (Valiente and Albrigo, 2004; Knauer *et al.*, 2011). Optimal flowering density is achieved only upon accretion of sufficient cool hours. It is assumed that a heavy fruit load prevents recognition of the low-temperature flowering inductive signal and/or blocks later stages of inflorescence, such as bud break (Albrigo and Galán-Saúco, 2004; Verreynne and Lovatt, 2009). As expected, fruit load affects the expression of flowering control genes, *FT*, *LFY*, *AP1*, *TFL*, and *miR156*-regulated *SQUAMOSA PROMOTER BINDING* (*SPL5*) in leaves and buds of citrus (Muñoz-Fambuena *et al.*, 2011, 2012*b*; Shalom *et al.*, 2012) as well as in mango and apple (Kotoda *et al.*, 2010; Nakagawa *et al.*, 2012).

The mechanism by which heavy crop load affects return bloom is not fully understood. The developing fruit provides a strong sink for photoassimilates. It was therefore thought that depletion of photoassimilates, especially carbohydrates from the bud, prevents flowering induction, a hypothesis known as the nutritional theory (Goldschmidt *et al.*, 1985; Goldschmidt, 1999). Sucrose was shown to play a regulatory role in *Arabidopsis* flowering control (Eriksson *et al.*, 2006), but whether sugars indeed play a regulatory role in flowering induction under various fruit loads in fruit trees has been a controversial issue for many years (Hilgeman *et al.*, 1967; Jones *et al.*, 1970, 1974; Goldschmidt and Golomb, 1982; Li *et al.*, 2003*a*, *b*). Recent work has shown that trehalose metabolism and its product trehalose-6-phosphate were involved in flowering control in *Arabidopsis* (van Dijken *et al.*, 2004; Wahl *et al.*, 2013). It was also shown that two genes encoding enzymes associated with trehalose metabolism were induced in OFF-Crop buds (Shalom *et al.*, 2012). In addition to the nutritional control of alternate bearing (AB), it might well be that the fruit itself, or an organ which senses fruit presence, generates an inhibitory signal (AB signal) which moves into the bud and prevents flowering induction (Bower *et al.*, 1990; Tálon *et al.*, 1997). Fruit thinning or complete removal (de-fruiting) from ON-Crop trees induces return bloom (Monselise and Goldschmidt, 1981), thus providing support for this notion. Gibberellin (GA) is known to inhibit flowering in many perennials (Goldschmidt and Samach, 2004; Bangerth, 2009). However, while exogenous application of GA prevents flowering (Goldschmidt *et al.*, 1997; Muñoz-Fambuena *et al.*, 2012*a*; Goldberg-Moeller *et al.*, 2013), the question of whether GA acts endogenously to inhibit flowering is still open. The involvement of abscisic acid (ABA) in the regulation of return bloom is even less clear (Jones *et al.*, 1976; Goldschmidt, 1984; Koshita *et al.*, 1999; Okuda, 2000). Polar auxin transport from a dominant sink was also suggested as a possible mobile signal affecting flowering (Caaejas and Bangerth, 1997; Smith and Samach, 2013).

Fruit load might act at various developmental stages such as flowering induction, transition of the shoot apical meristem, and subsequent stages of flower development and bud break (Verreynne and Lovatt, 2009). Regardless of the source of the AB signal and its nature, it must be recognized by its receptor in the bud which in turn must make the ‘decision’ of whether to proceed to inflorescence or not. In order to investigate metabolic and regulatory processes taking place in the bud and affected by fruit load, the transcriptome of buds from ON- and OFF-Crop trees was recently compared during three developmental stages. Changes in metabolic and regulatory pathways, including photosynthesis, and in flavonoid and trehalose metabolism were identified (Shalom *et al.*, 2012). However, this work was biased due to the use of an Affymetrix Citrus Gene-Chip array that contained ~15 500 genes. In fact, with the exception of trehalose metabolism, no other regulatory pathways were identified. In the current work, a complementary approach was taken by comparing the transcriptome of buds of de-fruited trees with those of ON-Crop trees. The genomic analysis was non-biased, as it was based on RNA-deep sequencing. It was possible to identify an increase in ABA-metabolizing genes, accompanied by a decrease in ABA levels and those of its catabolites in buds of de-fruited trees. Moreover, a remarkable increase in the expression of genes encoding proteins associated with calcium-dependent auxin polar transport and a reduction in bud endogenous auxin levels following de-fruiting were identified. The results are discussed in light of the previously suggested auxin transport autoinhibition (ATA) theory (Bangerth, 1989) and its role in AB (Smith and Samach, 2013).

## Materials and methods

### Plant material and sample collection

Plant material was collected from a commercial orchard of 15-year-old Murcott mandarin (*Citrus reticulate* Blanco) trees grafted on sour orange (*Citrus aurantium* L.), located in the central coastal area of Israel, during the years 2011 (an ON year) and 2012 (an OFF year). Although most of the trees in the orchard bore similar yields in a given year, some were aberrant and showed an opposite AB trend. These and nearby trees with the opposite yield status were selected. Overall, nine triplets of trees were chosen, with each triplet (two ON trees and a nearby OFF tree) being considered one biological replicate. Fruits were completely removed (de-fruiting) on 22 August 2011 from one of the ON trees in each triplet, and this tree was labelled DEF. Samples were collected 1 d prior to de-fruiting (Time 0, from ON and OFF trees), 1 week following de-fruiting (Time 1, from ON and DEF trees), 2 weeks following de-fruiting (Time 2, from ON and DEF trees), and 4 weeks following de-fruiting (Time 4, from ON, OFF, and DEF trees).

The three most extreme conditions of spring flush flowering were compared (see fig. S2 in Shalom *et al.*, 2012): fruit-bearing flush of an ON tree, fruitless flush of an OFF tree, and de-fruited flush of a DEF tree. Branches of each of these conditions were collected from the southeast side of the trees, taken to the laboratory on ice, and buds were separated and immediately frozen in liquid nitrogen. The percentages of generative, mixed, and vegetative shoots were determined for all branches splitting off from one major 50–60mm diameter branch located on the southeast side of the tree, during peak blossoming time (usually the first 10 d of April of the consecutive year). Selection of the branches to be sampled was done prior to bud break.

### RNA extraction, quantification, and qPCR analyses

Total RNA was extracted, treated, and analysed from ~0.2g of frozen bud tissue, and cDNA was synthesized, as previously described (Shalom *et al.*, 2012). Primers for the genes *CiFT2*, *CsLFY*, *SPL5*, *RbcS*, *LHCB3*, *PRK*, *PSB28*, *PSAD*, *SHM*, *Fd*, *NCED3*, *CAX*, *PBP1-like1*, *PBP1-like2*, *NPH3*, *CA-binding EF hand1*, *β-ACTIN*, and a dual-labelled probe for *CiFT2* were designed based on genomic and expressed sequence tag (EST) sequences (Phytozome, http://www.phytozome.net/; HarvEST, http://harvest.ucr.edu/) using Primer 3 software (Supplementary Table S1 available at *JXB* online). Real-time PCR was carried out as described (Shalom *et al.*, 2012). For the *CiFT2* dual-labelled probe reaction, real-time PCR was carried out as described by Shalom *et al.* (2012). The mRNA levels of trehalose biosynthetic genes and flavonoid biosynthetic genes (Supplementary Table S2) were determined by nCounter analysis (Nanostring Technologies, Seattle, WA, USA) at the VIB MicroArrays Facility (Leuven, Belgium), as described by Shalom *et al.* (2012).

### RNA deep sequencing

Extracted RNA integrity was determined by Agilent Bioanalyzer (Santa Clara, CA, USA) according to the manufacturer’s instructions. A 2 μg aliquot of total RNA from each sample was prepared and used for cDNA library constructions using the TruSeq mRNA sample preparation kit according to the manufacturer’s protocol (Illumina Inc., San Diego, CA, USA, REF 15025062). The libraries (10 pmol) were run on a single read 100 nucleotide run on the HiSeq 2000 (Illumina Inc.) on six lanes. Raw fastq files were quality checked using FastQC (http://www.bioinformatics.babraham.ac.uk/projects/fastqc), and adaptor sequences were removed using fastq-mcf (https://code.google.com/p/ea-utils/wiki/FastqMcf). They were then aligned to the orange (*Citrus sinensis*) genome database (Xu *et al.*, 2013; http://citrus.hzau.edu.cn/orange/) using TopHat2 (Kim *et al.*, 2013). FPKM (fragments per kilobase of transcript per million mapped reads) values, which normalize the read count by the length of the fragment and the total number of mapped reads, were calculated, and differential expression was checked using Cufflinks (Trapnell *et al.*, 2010). A hierarchical clustering heatmap and a 2D principle component analysis (2D PCA) plot were generated by MATLAB (Mathworks, Cambridge, UK), using the log-FPKM values of each gene. For gene ontology (GO) analysis, sequences were blasted against the UniRef90 database (Suzek *et al.*, 2007), by which a number of GO annotations were derived for each gene. Singular enrichment analysis (SEA), which lists enriched GO terms, was performed [false discovery rate (FDR) ≤0.05] for the five best GO terms of each gene using the AgriGo interface (http://bioinfo.cau.edu.cn/agriGO/index.php). Differentially expressed genes were also functionally annotated via the automated Mercator pipeline (Lohse *et al.*, 2013) (http://www.gabipd.org/biotools/mercator/) and displayed on diagrams of metabolic and other processes using MapMan (Usadel *et al.*, 2009; http://www.gabipd.org/projects/MapMan/).

### Hormonal content analysis

About 50mg of bud tissue from each tested sample were lyophilized and homogenized to a fine powder in liquid nitrogen using a mortar and pestle. Quantification of ABA, ABA metabolites, and indole acetic acid (IAA) was conducted at the National Research Council of Canada (Saskatoon, Saskatchewan, Canada) according to published protocols (http://www.nrc-cnrc.gc.ca).

### Protein extraction and western blot analysis

Proteins were extracted from buds, quantified, and analysed by western blot analysis using specific primary antibodies, raised against the following proteins: Rubisco complex large (RbcL) and small (RbcS) subunits, ferredoxin (Fd), chlorophyll *a/b* protein [light-harvesting chlorophyll *a/b* complex II (LHCII)], and D1 protein (PsbA), as described by Maayan *et al.* (2008).

### Statistical analysis

The statistical analyses used for qPCR results, hormone analyses, and inflorescences numbers were one-way analysis of variance (ANOVA) with Tukey–Kramer multiple comparison tests as implemented in the software JMP version 10 (SAS Institute).

## Results

### Fruit removal (de-fruiting) induces back flowering

**Fig. 1.**
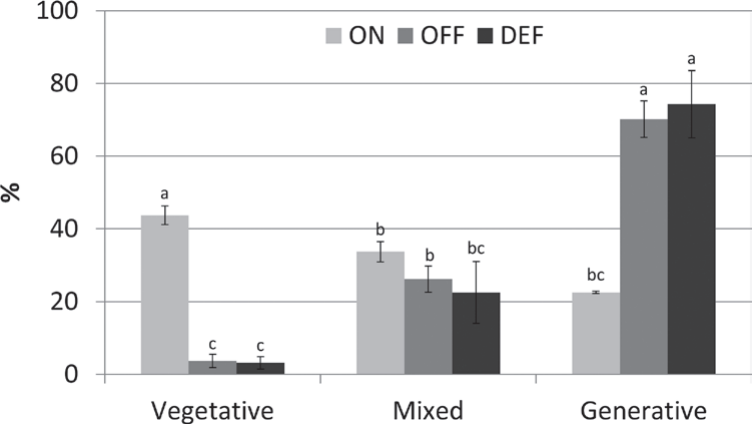
Fruit load affects flowering intensity the following year. Vegetative shoots, generative inflorescences containing only flower buds, and mixed inflorescences containing flower buds and leaves in various ratios were counted during flowering peak in trees which carried a heavy yield (ON) or light yield (OFF), and in de-fruited trees (DEF). The numbers are mean values of three independent biological replicates ±SE. Different letters represent a significant difference (*P*≤0.05).

Normally, fruit load status is similar among most trees of the orchard—trees bear either a heavy crop (ON-Crop year) or a low crop (OFF-Crop year). A few trees, however, show the opposite trend, allowing the collection of samples from nearby trees bearing either high fruit load or low fruit load. In order to detect changes which might play a role in converting ON- to OFF-Crop buds, fruits were completely removed from ON-Crop trees in August and buds were collected 1, 2, and 4 weeks following de-fruiting. The effect of the treatment was verified the following spring by counting the number of inflorescences and vegetative shoots (Fig. 1). Citrus trees bear two major types of inflorescences: generative (leafless) and mixed (leafy; flowers and leaves in various ratios). ON-Crop trees had significantly less generative inflorescences compared with OFF-Crop trees and DEF trees (22% versus 70–75%) and more vegetative shoots (43% versus 3–4%). As expected, no significant differences were detected in mixed-type inflorescences. Fruit counting during harvest time showed that ON-Crop trees yielded 1635±98 fruits per tree while OFF-Crop trees yielded 36±12 fruits per tree.

### Fruit removal alters expression of flowering control and other genes in the bud

**Fig. 2.**
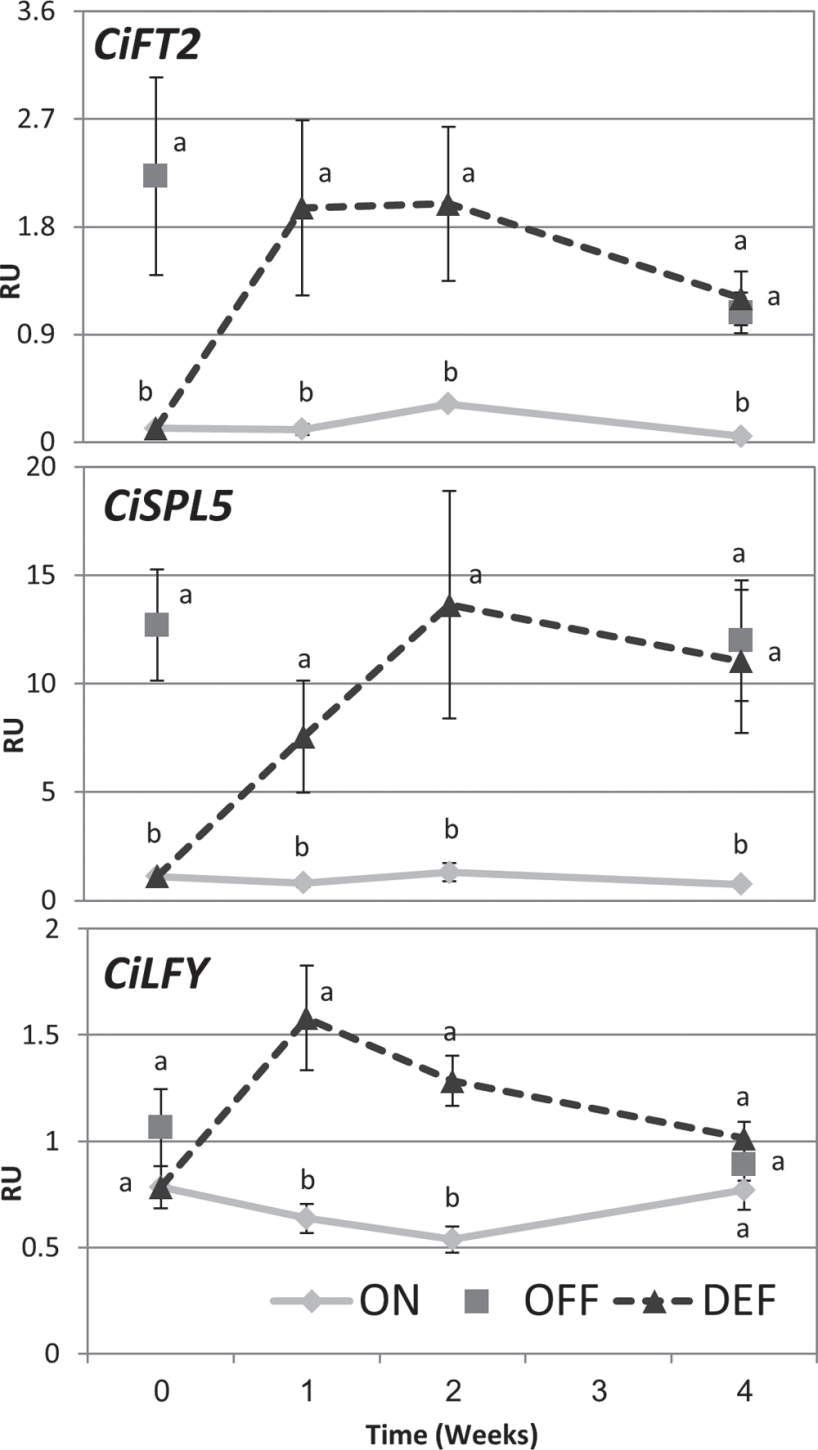
Fruit removal alters the expression of flowering control genes in buds. The mRNA levels (RU, relative units) of the indicated genes were determined in ON-Crop (ON), OFF-Crop (OFF), and de-fruited (DEF) trees at the indicated weeks following de-fruiting. The numbers are mean values of three independent biological replicates ±SE. Different letters represent a significant difference (*P*≤0.05) between the states at the same time point.

In order to determine how quickly buds responded to de-fruiting, the mRNAs levels of several flowering control genes were quantified during the course of the experiment (Fig. 2). The *Citrus* genome contains three *FT* genes, but only the expression of *CiFT2* correlated well with tree flowering intensity (Nishikawa *et al.*, 2007; Shalom *et al.*, 2012). In buds of ON-Crop trees, *CiFT2* mRNA levels were relatively low, and remained unchanged. In contrast, *CiFT2* mRNA was ~15-fold higher in buds of OFF-Crop trees. One week following de-fruiting, the expression of the gene in the buds of the DEF trees was similar to that of OFF-Crop buds, and it remained at this level during the entire test period. The mRNA levels of *LFY* were similar in buds of ON- and OFF-Crop trees, but it increased 3-fold within 1 week in DEF buds and returned to its basal level after 4 weeks. It was previously shown that *miR156*-regulated *SPL5* displayed elevated mRNA levels in OFF-Crop buds; thus, it may act as a positive inducer of flowering in *Citrus* trees (Shalom *et al.*, 2012). As expected, *SPL5* mRNA levels were ~14-fold higher in buds of OFF-Crop trees as compared with ON-Crop buds. DEF buds displayed increased mRNA as compared with OFF-Crop buds within 2 weeks of de-fruiting. The expression of genes associated with trehalose and flavonoid metabolism was elevated in ON- and OFF-Crop buds (Shalom *et al.*, 2012). As expected, the mRNA levels of trehalose phosphate synthase and trehalose phosphate phosphatase were reduced by 2- and 4-fold, respectively following de-fruiting (Supplementary Fig. S1 at *JXB* online). The mRNA levels of flavonoid biosynthesis genes, *UF3GT*, *4CL*, *CHS*, and *CHI*, were induced by the treatment. De-fruiting resulted in a reduction of *C4H* mRNA levels in the ON-Crop trees.

### Fruit removal induces rapid changes in the bud global gene expression

**Fig. 3.**
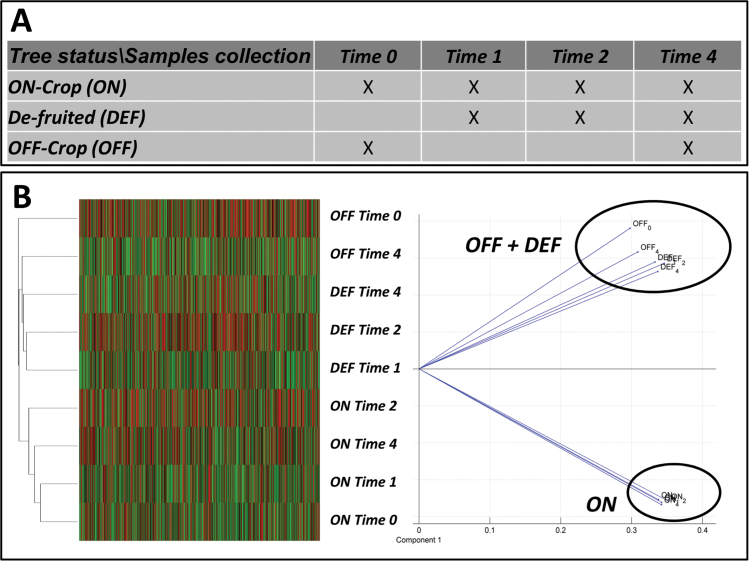
Global gene expression in buds of de-fruited (DEF) trees is similar to that of buds of OFF-Crop (OFF) trees. The experimental design and sample collection (A). Hierarchical clustering heatmap (left panel) and 2D principle component analysis (right panel) plots were generated using the log-*FPKM* value of each gene for all samples, as indicated in A (B). (This figure is available in colour at *JXB* online.)

**Fig. 4.**
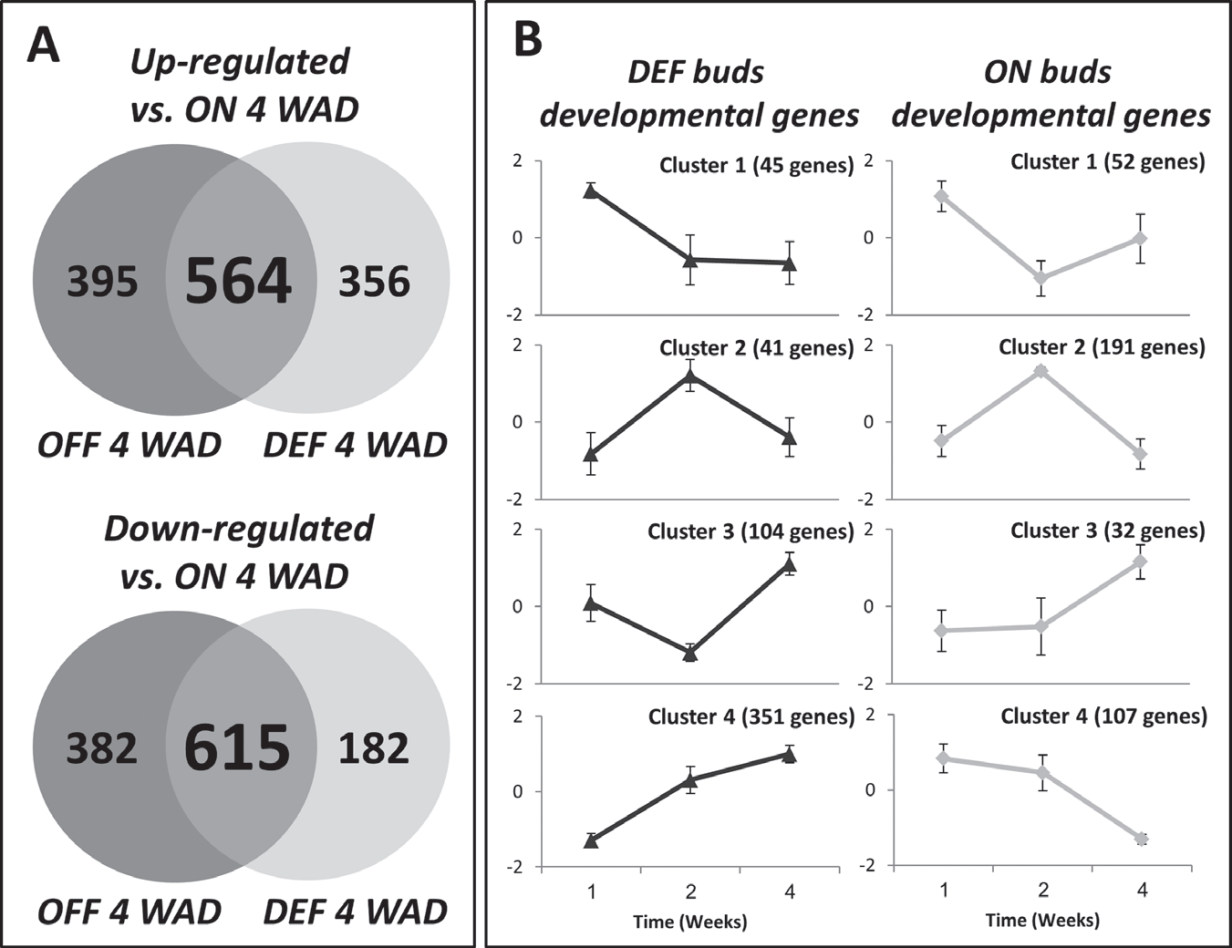
Venn diagrams of differentially expressed genes and clustering analysis of developmentally altered genes. The number of genes in buds of OFF-Crop (OFF) and de-fruited (DEF) trees which were significantly (*P*≤0.05) up-regulated (left) or down-regulated (right) compared with ON-Crop buds at Time 4 (A). Clustering analysis of genes which were significantly (*P*≤0.05) altered developmentally during the course of the experiment in comparison with Time 0 as generated by Expender (http://acgt.cs.tau.ac.il/expander/overview.html) using the Click Algorithm (B). All genes are listed in Supplementary File 2 at *JXB* online.

The results indicated that the transition of the bud from an ON to an OFF state took place relatively quickly. In order to analyse the metabolic and regulatory pathways playing a role in this transition, changes in global gene expression were analysed in buds before de-fruiting (Time 0) and 1 (Time 1), 2 (Time 2), and 4 (Time 4) weeks after de-fruiting (Fig. 3A). In addition, buds of OFF-Crop trees were also analysed at Time 0 and Time 4. The minimum number of reads per sample was ~15 million and the maximum was ~40 million, indicating a deep and satisfactory coverage of the existing transcripts (Supplementary Table S3 at *JXB* online). Overall, the number of transcripts in all libraries was ~14 400. Hierarchical cluster analysis (Fig. 3B, left) and 2D PCA (Fig. 3B, right) were performed based on the log-FPKM values of each sample. Results of both analyses showed that during all time points, including Time 1, the transcript profiles of DEF buds were more closely related to those of OFF-Crop buds than to those of ON-Crop buds, thus supporting the notion that the transition from an ON bud to an OFF bud was relatively quick following de-fruiting. In order to analyse the metabolic and regulatory pathways mediating the ON bud to OFF bud transition, two major comparisons were made (the ratios for all possible comparisons in the experiment are presented in Supplementary File 2 at *JXB* online). First, Time 4 included the three fruit load states, ON, OFF, and DEF. Therefore, the genes that were up- or down-regulated (*P*≤0.05) in OFF or DEF buds in comparison with ON-Crop buds were identified (Fig. 4A; Supplementary File 2). Overall, 997 genes in OFF-Crop buds and 797 genes in DEF buds were down-regulated relative to ON-Crop buds at Time 4, with 615 genes common to the two groups (OFF and DEF). Overall, 959 genes in OFF-Crop buds and 920 genes in DEF buds were up-regulated relative to ON-Crop buds at Time 4, with 564 genes common to the two groups (OFF and DEF) (Fig. 4A; Supplementary File 2). The second comparison aimed at identifying genes which showed alternation in their expression during the course of the experiment and also common or different pathways altered developmentally. For that, genes were clustered according to their expression patterns relative to Time 0. For ON-Crop and DEF buds, four clusters were identified (Fig. 4B; Supplementary File 2). For OFF-Crop buds, only two time points were analysed, and altered genes were either reduced or repressed (Supplementary File 2)

In order to identify common and unique metabolic and regulatory pathways in DEF and OFF-Crop buds which were altered in comparison with ON-Crop buds at Time 4, up-regulated genes (564+395 and 564+356, Fig. 4A) and down-regulated genes (615+382 and 615+182, Fig. 4A) were GO annotated and further analysed using SEA (AgriGO). Table 1 presents these common processes. However, in a few cases, unique GO terms were included under the same general process either because of redundancy or because they were part of one process. The analysis indicated that the great majority of common processes which were enriched in DEF/OFF-Crop buds were related to light sensing (such as response to light stimulus/intensity and response to far red/blue and red light), photosynthesis (such as dark and light reactions and photosynthetic electron transport chain), chloroplast reorganization (such as plastid localization, chloroplast relocation and organization), response to carbohydrate stimulus (i.e. sucrose and disaccharide), ion homeostasis (such as cation homeostasis and transport, proton transport), and the pentose-phosphate cycle. Fewer genes were down-regulated in DEF/OFF-Crop buds and belonged to several secondary metabolic pathways such as the terpenoid and phenylpropanoid metabolic process, and oxidative reduction. Major unique processes induced in OFF-Crop buds included starch biosynthesis, carbohydrate metabolism, glycoside and glycosinolate metabolism, and water homeostasis (Supplementary Table S4 at *JXB* online). Considerably more unique processes were induced in DEF buds as compared with OFF-Crop buds (Supplementary Table S4). They included regulation of peptidase activity, regulation of dephosphorylation, and salicylic acid metabolism. Down-regulated unique processes in DEF buds included amino acid/amine metabolism and aromatic compound metabolism (Supplementary Table S4). Responses to biotic stress were either induced or repressed in DEF buds. Unique genes down-regulated in OFF-Crop buds could not be GO annotated, due to their low number.

**Table 1.** Gene ontology categorization of common genes up- or down-regulated in buds of OFF-Crop and de-fruited (DEF) trees relative to buds of ON-Crop trees at Time 4

General process	GO term	Description	Ref item	OFF				DEF			
				Query item	*P*-value	FDR	Fold enrichment^*a*^	Query item	*P*-value	FDR	Fold enrichment^*a*^
Up-regulated											
Response to light	GO:0010218	Response to far red light	25	11	2.80E-07	0.00057	6.48	14	7.20E-11	9.40E-08	8.51
	GO:0010114	Response to red light	47	14	1.90E-06	0.00089	4.39	17	3.80E-09	3.00E-06	5.50
	GO:0009639	Response to red or far red light	r	22	0.00023	0.024	2.31	22	0.00015	0.014	2.39
	GO:0009416	Response to light stimulus	380	44	0.00055	0.047	1.70	59	2.20E-09	2.20E-06	2.36
	GO:0009637	Response to blue light	27	7	8.20E-05	0.049	6.39	11	5.20E-07	0.00015	6.19
	GO:0009644	Response to high light intensity	70					20	1.80E-08	8.90E-06	4.34
	GO:0009642	Response to light intensity	88					23	1.10E-08	7.00E-06	3.97
	GO:0009314	Response to radiation	404					59	2.10E-08	9.30E-06	2.22
Photosynthesis and carbon fixation	GO:0015977	Carbon fixation	7	6	6.60E-07	0.00068	12.62	5	2.40E-05	0.0031	10.85
	GO:0009773	Photosynthetic electron transport in photosystem I	12	7	4.00E-06	0.0013	8.59	5	0.00067	0.038	6.33
	GO:0015979	Photosynthesis	128	33	2.90E-11	1.20E-07	3.80	47	9.80E-23	3.90E-19	5.58
	GO:0009767	Photosynthetic electron transport chain	31	10	2.60E-05	0.0059	4.75	11	2.60E-06	0.00055	5.39
	GO:0019684	Photosynthesis, light reaction	85	19	3.90E-06	0.0013	3.29	31	1.60E-15	3.10E-12	5.54
	GO:0019685	Photosynthesis, dark reaction	5	5	1.50E-06	0.00087	14.72				
	GO:0010207	Photosystem II assembly	36					13	2.60E-07	8.50E-05	5.49
	GO:0009765	Photosynthesis, light harvesting	13					8	3.50E-07	0.00011	9.35
Chloroplast	GO:0051667	Establishment of plastid localization	28	9	6.70E-05	0.011	4.73	8	0.00034	0.023	4.34
	GO:0051644	Plastid localization	28	9	6.70E-05	0.011	4.73	8	0.00034	0.023	4.34
	GO:0009902	Chloroplast relocation	28	9	6.70E-05	0.011	4.73	8	0.00034	0.023	4.34
	GO:0051656	Establishment of organelle localization	31	9	0.00016	0.02	4.27	8	0.00073	0.04	3.92
	GO:0009658	Chloroplast organization	81	18	7.60E-06	0.002	3.27	14	0.00083	0.044	2.63
	GO:0009657	Plastid organization	123	21	9.50E-05	0.014	2.51	20	0.00018	0.015	2.47
Response to carbohydrate stimulus	GO:0009744	Response to sucrose stimulus	47	12	5.60E-05	0.011	3.76	14	1.30E-06	0.00033	4.53
	GO:0034285	Response to disaccharide stimulus	48	12	7.10E-05	0.011	3.68	14	1.80E-06	0.00039	4.43
	GO:0009743	Response to carbohydrate stimulus	178	31	1.70E-06	0.00088	2.56	33	9.30E-08	3.30E-05	2.82
Cellular homeostasis	GO:0071704	Organic substance metabolic process	7	6	6.60E-07	0.00068	12.62	5	2.40E-05	0.0031	10.85
	GO:0019725	Cellular homeostasis	129	23	2.30E-05	0.0055	2.63	21	0.00012	0.012	2.47
	GO:0055082	Cellular chemical homeostasis	57	12	2.90E-06	0.0037	5.19	16	5.90E-07	0.00016	4.26
Ion homeostasis	GO:0006873	Cellular ion homeostasis	52	12	0.00016	0.02	3.40	14	5.00E-06	0.00095	4.09
	GO:0030003	Cellular cation homeostasis	50	11	0.00046	0.043	3.24	13	1.60E-05	0.0023	3.95
	GO:0055082	Cellular chemical homeostasis	57	15	4.70E-06	0.0014	3.87	41	0.00013	0.012	1.87
	GO:0006812	Cation transport	333	43	6.00E-05	0.011	1.90	14	0.00037	0.023	2.84
	GO:0006811	Ion transport	470	55	0.0001	0.014	1.72	52	0.00028	0.021	1.68
	GO:0010155	Regulation of proton transport	11	6	3.40E-05	0.0075	8.03	7	1.40E-06	0.00033	9.67
	GO:0055080	Cation homeostasis	64					13	0.00025	0.019	3.09
Pentose cycle	GO:0019253	Reductive pentose-phosphate cycle	5	5	1.50E-06	0.00087	14.72				
	GO:0006098	Pentose-phosphate shunt	65					13	0.0003	0.022	3.04
Response to biotic stress	GO:0010200	Response to chitin	90	16	0.00038	0.038	2.62	17	8.10E-05	0.0089	2.87
	GO:0050832	Defence response to fungus	123	20	0.00027	0.028	2.39	20	0.00018	0.015	2.47
Abiotic stress	GO:0009611	Response to wounding	128	20	0.00046	0.043	2.30	20	0.00031	0.022	2.37
Down-regulated											
Secondary metabolism	GO:0019748	Secondary metabolic process	467	58	5.80E-05	0.042	1.73	55	1.20E-06	0.0011	2.04
	GO:0006721	Terpenoid metabolic process	118	23	1.20E-05	0.016	2.72	18	0.00017	0.028	2.64
	GO:0006720	Isoprenoid metabolic process	186	29	8.10E-05	0.049	2.17	8	0.00031	0.044	4.46
	GO:0009699	Phenylpropanoid biosynthetic process	143					23	1.00E-05	0.0035	2.78
	GO:0009698	Phenylpropanoid metabolic process	184					27	1.10E-05	0.0035	2.54
Various	GO:0055114	Oxidation reduction	1344	166	2.00E-10	7.40E-07	1.72	137	2.60E-09	7.50E-06	1.76
	GO:0042221	Response to chemical stimulus	1345	142	1.30E-05	0.016	1.47	120	1.00E-05	0.0035	1.54
	GO:0010035	Response to inorganic substance	462	58	4.30E-05	0.039	1.75				

^*a*^ Fold enrichment as calculated based on GO-annotated genes (777, 753, 821, 662 in OFF-Crop up-regulated, DEF up-regulated, OFF-Crop down-regulated, DEF down-regulated, respectively) in the list of genes (959, 920, 997, 797 in OFF-Crop up-regulated, DEF up-regulated, OFF-Crop down-regulated, DEF down-regulated, respectively) per GO-annotated genes (11445) in the reference list (14400 genes).

GO annotation and SEA were also performed to developmentally altered genes compared with Time 0 in ON-Crop, OFF-Crop, and DEF buds (Fig. 4B). Significantly altered biological processes (FDR ≤0.05) could be identified for genes of clusters 3 and 4 (DEF buds), cluster 2 (ON-Crop buds), and up/down regulated genes of OFF-Crop buds. No significant biological processes could be identified even under FDR ≤0.1 in genes of the other clusters. Cluster 4 of DEF buds included processes involved in light responses and photosynthesis (Supplementary Table S5 at *JXB* online). A considerable induction was also detected in genes of respiratory burst and in those involved in responses to abiotic stresses. Genes up-regulated in OFF-Crop buds from Time 0 to Time 4 were annotated into light responses, photosynthesis, and response to carbohydrate stimulus (Supplementary Table S6). Down-regulated genes in OFF-Crop buds from Time 0 to Time 4 included responses to jasmonic acid (JA) and ethylene (Supplementary Table S7). Other processes included amino acid and amine metabolism, and responses to abiotic stresses. Cluster 3 of DEF buds included 104 genes that could be annotated into different biological processes, a few of them related to development and morphogenesis, such as trichoblast and root differentiation, floral organ development, and shoot morphogenesis (Supplementary Table S8). Cluster 2 of ON-Crop buds was enriched in genes associated with lipid transport (Supplementary Table S9).

An additional analysis for developmentally regulated genes was aimed at identifying common genes in clusters showing a similar expression pattern in OFF-Crop and DEF buds and opposite patterns in ON-Crop buds. The first comparison included cluster 4 of DEF buds, up-regulated genes of OFF-Crop buds, and cluster 4 of ON-Crop buds (Supplementary Fig. S2A at *JXB* online). However, only one unknown gene was common among the three states. The second comparison included cluster 1 of DEF buds, down-regulated genes of OFF-Crop buds, and cluster 3 of ON-Crop buds (Supplementary Fig. S2B). Only the expression of three genes was common; two of them were homologous to terpene (nerolidol) syntheses (*Cs2g07240* and *Cs2g07250*).

### Photosynthetic genes and proteins are up-regulated in response to fruit removal

**Fig. 5.**
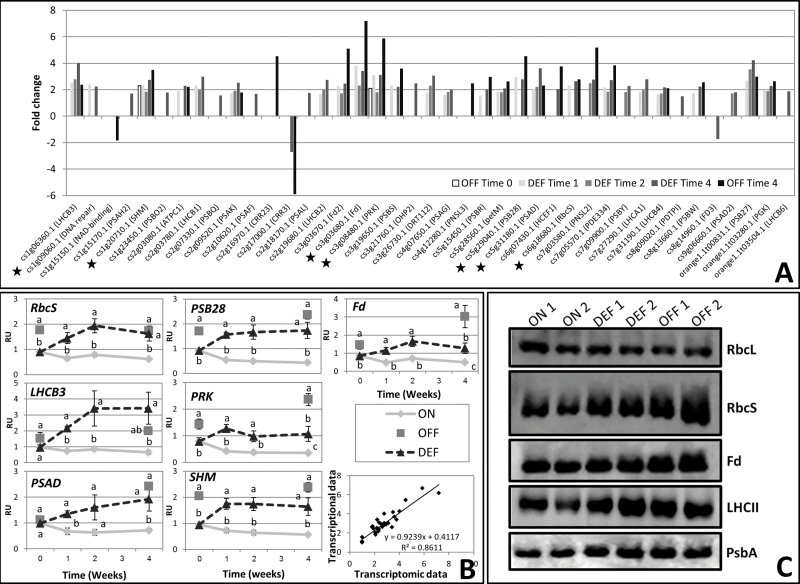
The photosynthetic machinery is up-regulated in buds of OFF-Crop (OFF) and de-fruited (DEF) trees. Fold-change (*P*≤0.05) in the expression of photosynthetic genes (determined by MapMan analysis, see Supplementary Fig. S2 at *JXB* online) in buds of OFF and DEF trees relative to buds of ON-Crop (ON) trees at the indicated time points. Asterisks mark genes selected for validation by qPCR analyses, and specific genes are listed in Supplementary File 2 (A). Expression analysis of selected genes at the indicated weeks following de-fruiting, as determined by qPCR analyses (RU, relative units). The numbers are mean values of three independent biological replicates ±SE. Different letters represent a significant difference (*P*≤0.05) between the states at the same time point. The lower right graph shows the linear regression between the transcriptomic and transcriptional (qPCR) data (B). Immunoblot analyses in two independent replicates of photosynthetic proteins (RbcL, Rubisco large subunit; RbcS, Rubisco small subunit; Fd, ferredoxin; LHCII, light-harvesting complex II; Psba, D1 protein) extracted from two replicates of buds of ON-Crop, (ON1 and ON2), OFF-Crop (OFF1 and OFF2), and de-fruited (DEF1 and DEF2) trees at Time 4 (C). The quantification of the protein signals, generated using ImageJ software, is presented in Supplementary Fig. S4.

Consistent with a previous report (Shalom *et al.*, 2012), the above-described results and MapMan analysis of differentially expressed genes in DEF versus ON-Crop buds at Time 4 showed that genes associated with light reactions, the Calvin–Benson cycle, and to a lesser extent photorespiration were induced (Supplementary Fig. S3 at *JXB* online). Fold changes of 41 photosynthetic genes in DEF and OFF-Crop buds relative to ON-Crop buds are shown in Fig. 5A. Out of 41 differentially expressed genes, 38 were up-regulated while only three were down-regulated at at least one time point throughout the experiment. Validation of the above results by qPCR analyses was performed for seven genes, *Ribulose bisphosphate carboxylase-small subunit* (*RbcS*), *Light-harvesting chlorophyll B-binding protein* (*LHCB3*), *Phosphoribulokinase* (*PRK*), *Photosystem II reaction centre PSB28* (*PSB28*), *Photosystem I subunit D* (*PSAD*), *Serine hydroxymethyltransferase* (*SHM*), and *Ferredoxin* (*Fd*) (Fig. 5B). The mRNA levels of all these genes were significantly higher in buds of OFF trees as compared with ON trees at Time 4. As expected, gene expression increased in DEF buds by 2.6- to 5.2-fold within 1 week of fruit removal. Furthermore, western blot analyses using specific antibodies raised against RbcS, Fd, LHCII, and PsbA showed that their protein level was higher in OFF-Crop buds relative to ON-Crop buds, and increased in DEF buds (at Time 4) following fruit removal (Fig. 5C). However, RbcL protein levels remained unchanged in DEF and OFF-Crop buds relative to ON-Crop buds.

### Fruit removal-induced changes in ABA-metabolizing genes

**Fig. 6.**
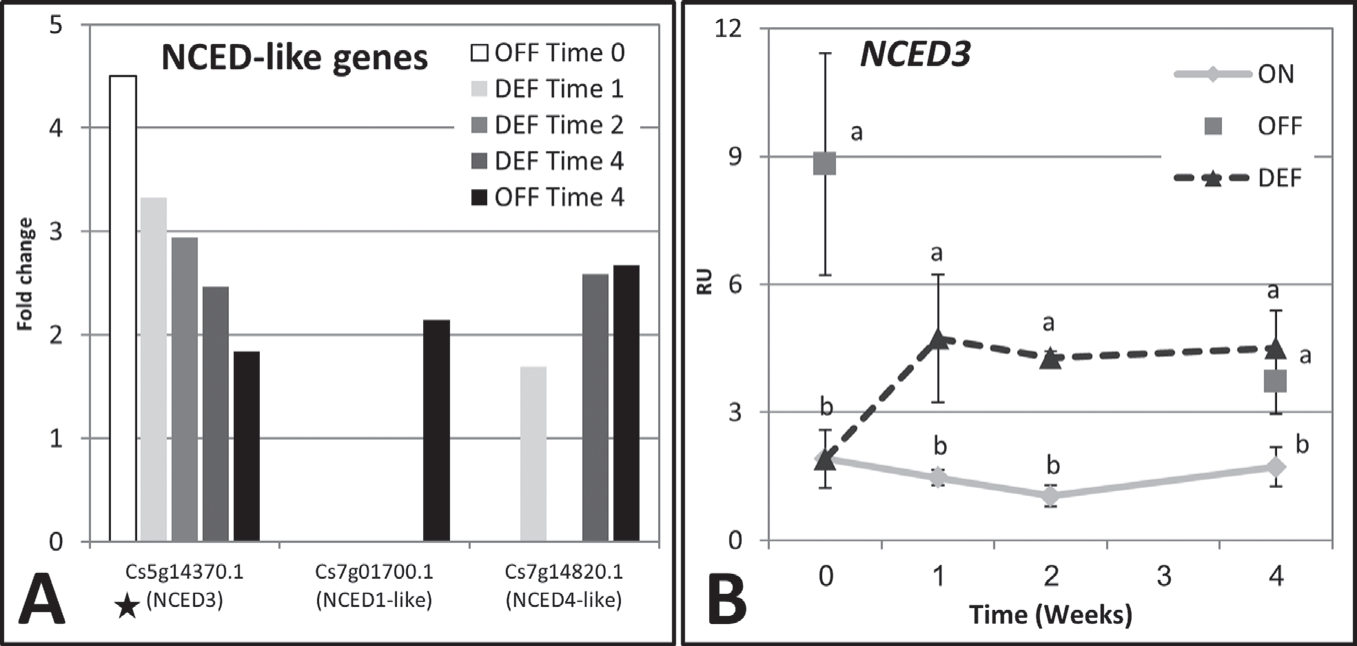
*NCED*-like genes are induced in buds of de-fruited (DEF) trees. Fold change (*P*≤0.05) in the expression of three *NCED-like* genes in buds of OFF-Crop (OFF) and DEF trees relative to buds of ON-Crop (ON) trees (an asterisk marks the *NCED3-like* gene which was validated by qPCR) (A). Expression of the *NCED3-like* gene in buds of ON, OFF, and DEF trees as determined by qPCR analyses at the indicated weeks following de-fruiting (RU, relative units). The numbers are mean values of three independent biological replicates ±SE (B). Different letters represent a significant difference (*P*≤0.05) between the states at the same time point.

Results of genomic analysis showed that genes homologous to *9-cis-epoxycarotenoid dioxygenase* (*NCED*), coding for this rate-limiting enzyme of ABA biosynthesis (Supplementary Fig. S5 at *JXB* online), were higher in buds of OFF-Crop buds relative to ON-Crop buds (Fig. 6A). In *Arabidopsis*, the *NCED* gene family comprises nine members, and the roles of *NCED2*, *3*, *5*, and *6* in ABA biosynthesis were demonstrated (Tan *et al.*, 2003). The *Citrus* genome contained nine highly homologous genes, and changes in three of them were detected in genomic analysis: *Cs5g14370.1*, homologous to *NCED3*, *Cs5g14370.1*, homologous to *NCED1*, and *Cs7g14820.1*, homologous to *NCED4*. Among these three genes, *NCED3-like* induction was increased by ~4-fold in OFF-Crop buds relative to ON-Crop buds at Time 0, and was ~2-fold higher at Time 4. Fruit removal induced a 3-fold increase at Time 1 and its mRNA level remained higher relative to ON-Crop buds throughout the experiment. Induction of *NCED1-like* was seen only in OFF-Crop buds at Time 4, while *NCED4-like* was induced in DEF buds at Time 1 and 4 and in OFF-Crop buds at Time 4. Among the three genes, NCED3 is considered the major enzyme catalysing the rate-limiting step in ABA biosynthesis, and the mRNA levels of its *Citrus* counterpart were higher in OFF and DEF buds throughout the experiment. qPCR validation showed that *Cs5g14370.1* mRNA levels in OFF-Crop buds were significantly higher relative to ON-Crop buds, by ~4.5-fold at Time 0 and by 2.1-fold at Time 4 (Fig. 6B). The mRNA levels in DEF buds increased relative to ON-Crop buds by 3.2-fold at Time 1 and remained higher throughout the experiment. The transcriptomic data showed that the expression of a gene homologous to *PYR1*, a component of the ABA receptor, was reduced (Supplementary Fig. S6).

**Fig. 7.**
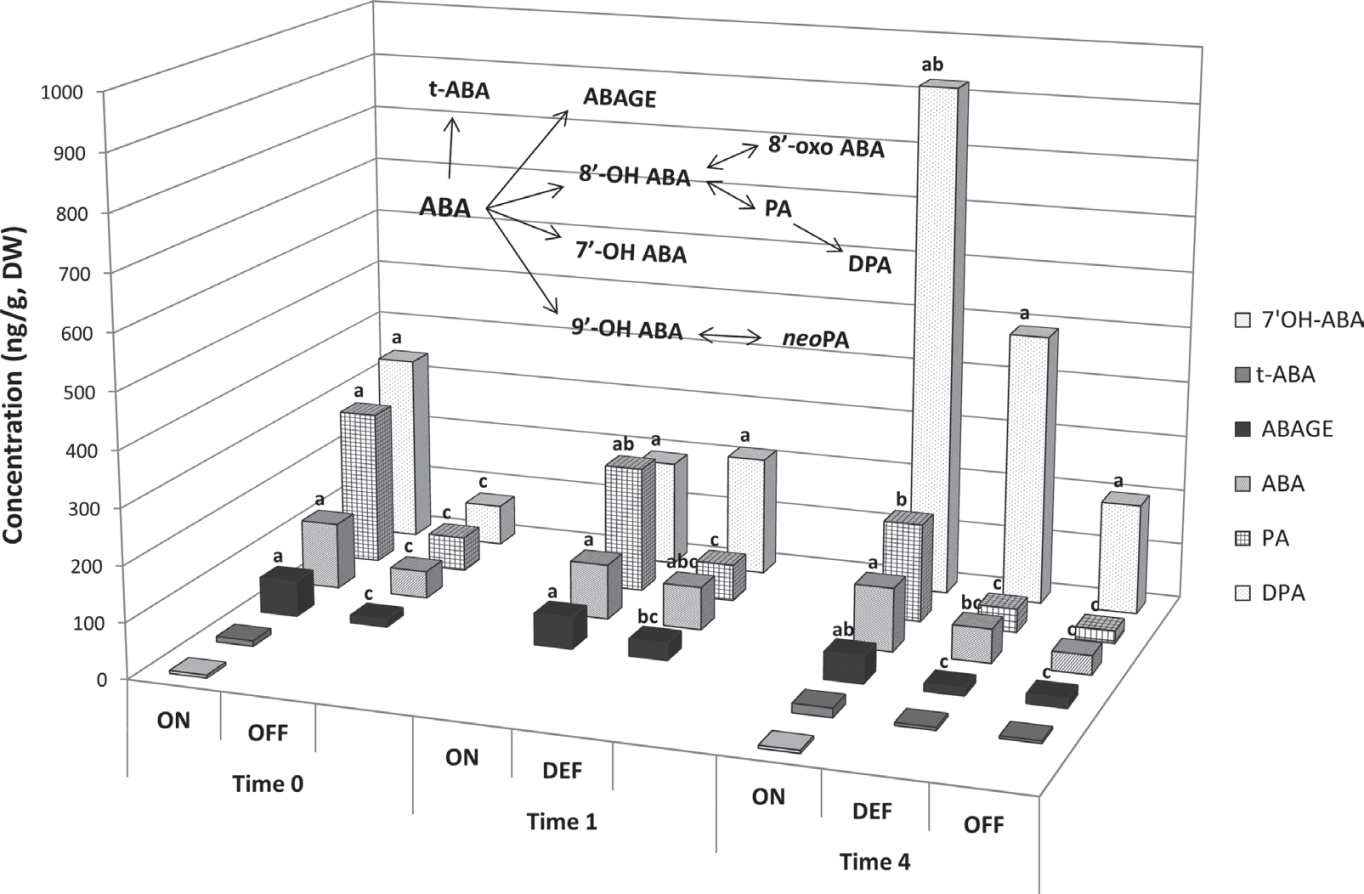
Fruit load affects the levels ABA and its catabolites in the bud. ABA and ABA catabolites (7’OH ABA, t-ABA, *trans*-ABA; ABAGE, ABA glucose ester; PA, phaseic acid; DPA, di-hydrophaseic acid) whose metabolic relationships are schematically represented in the inner scheme were determined at the indicated time points in buds of ON-Crop (ON), OFF-Crop (OFF), and de-fruited (DEF) trees. Means followed by different letters are significantly different (*P*>0.05) according to Tukey–Kramer multiple comparisons tests.

The levels of ABA and its catabolites were analysed at three time points following de-fruiting (Fig. 7). In addition to ABA and its isomer, *trans*-ABA (t-ABA), four catabolites were detected in the buds, 7’OH-ABA, ABA glucose ester (ABAGE), phaseic acid (PA), and dihydrophaseic acid (DPA), with t-ABA, PA, and ABAGE showing relatively high levels. ABA levels in ON-Crop buds were significantly higher relative to OFF-Crop buds, by 2.4-fold at Time 0 and by 3.3-fold at Time 4. In DEF buds, ABA levels decreased relative to ON-Crop buds by 1.3-fold at Time 1 and by 1.8-fold at Time 4. In addition, the levels of PA and ABAGE were significantly higher in ON-Crop buds relative to OFF-Crop buds by 4.5-fold and 4.6-fold at Time 1 and by 7.7-fold and 2.9-fold at Time 4, respectively. In DEF buds, PA and ABAGE levels decreased by 3.4-fold and 1.8-fold at Time 1 and by 4.1-fold and 3.5-fold at Time 4, respectively. The levels of DPA and t-ABA were generally higher in ON-Crop buds relative to OFF and DEF buds, especially at Time 4, but the differences were not significant.

### Induction of calcium-related genes associated with auxin transport in DEF and OFF-Crop buds

**Fig. 8.**
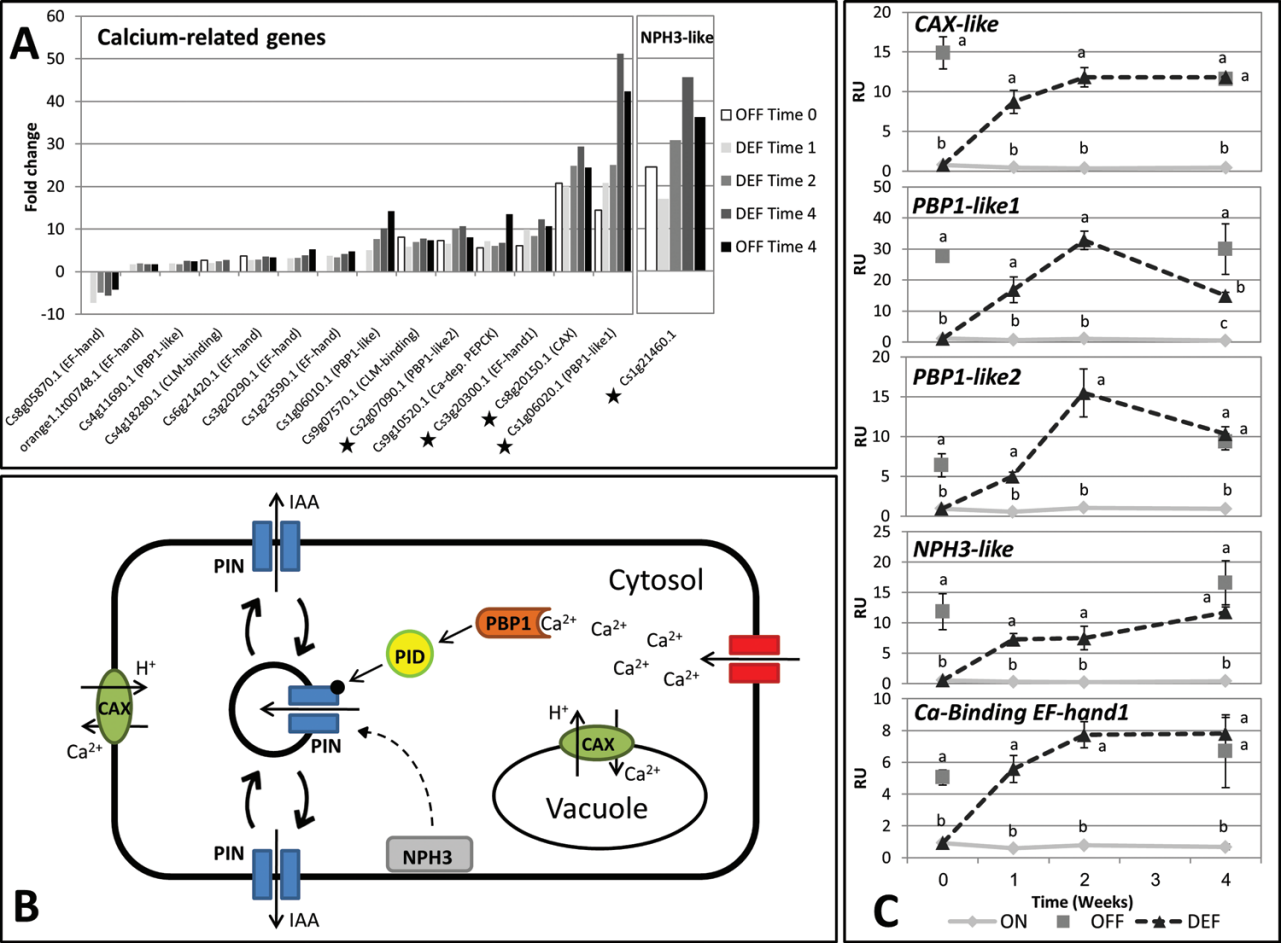
Fruit load affects the expression of Ca^2+^-related auxin polar transport and *NPH3-like* genes. Fold change (*P*≤0.05) in the expression of Ca^2+^-related and *NPH3-like* genes in buds of OFF-Crop (OFF) and de-fruited (DEF) trees relative to buds of ON-Crop (ON) trees (asterisks mark genes selected for validation by qPCR analyses; all genes are listed in Supplementary File 2 at *JXB* online) (A). Expression of the indicated genes in buds of ON, OFFm and DEF trees as validated by qPCR at the indicated time points following de-fruiting (RU, relative units). The numbers represent mean values of three independent biological replicates ±SE. Different letters represent a significant difference (*P*≤0.05) between the states at the same time point. (B) Linear regression between transcriptomic data and transcriptional data (qPCR analyses) is presented in Supplementary Fig. S7. Schematic model representing Ca^2+^ and NPH3 regulation of PIN cellular localization (C). (This figure is available in colour at *JXB* online.)

Among the genes which showed relatively high levels of expression in OFF-Crop and DEF buds relative to ON-Crop buds were calcium-related genes (Fig. 8A; Supplementary File 2 at *JXB* online). The expression of most of these genes was induced 3- to 40-fold at all time points of the experiment. Many of these genes encode proteins containing an EF-hand domain. PINOID (PID)-binding protein (PBPs) are a subgroup of EF-hand proteins. PBP1 has been shown to play a role in auxin polar transport in response to changes in calcium levels (Benjamins *et al.*, 2003). Four citrus *PBP1-like* genes (out of the five found in the citrus genome) were induced in DEF and OFF-Crop buds relative to ON-Crop buds at all time points tested (Fig. 8A). Phylogenic analysis of these genes showed remarkable homology with *PBP1* and its closely related gene in *Arabidopsis* (Supplementary Fig. S8A). Two other induced genes, *Cs9g20300.1* and *Cs8g20150.1*, showed high homology (80% and 70%, respectively) to a calcium-dependent protein kinase (*At1g08650.1*), and to a Ca^2+^/H^+^ antiporter *CAX3* (*At3g51860*), respectively. Another gene, *Cs1g21460.1*, which showed homology to members of the *NPH3* gene family from *Arabidopsis* also plays a role in auxin polar transport (Furutani *et al.*, 2011; Knauer *et al.*, 2011; Li *et al.*, 2011; Wan *et al.*, 2012). This family contains 33 genes in the *Arabidopsis* genome, and the *Citrus* genome comprises 25 homologous genes with relatively close taxonomic relationships (Supplementary Fig. S8B). The *NPH3-like* gene was induced 25- to 40-fold in OFF-Crop and DEF buds relative to ON-Crop buds at all time points tested (Fig. 8A). The plausible mechanistic relationships between calcium, PBP1, NPH3, and the polar subcellular localization of PIN-FORMED (PIN) auxin efflux carriers are schematically presented in Fig. 8B. The expression levels of *CAX-like*, *NPH3-like*, two *PBP1-like* genes, and one Ca^2+^-binding EF-hand gene were validated by qPCR analyses (Fig. 8C). The mRNA levels of all these genes were relatively low in ON-Crop buds and remained low throughout the experimental period. In OFF-Crop buds, they were significantly higher at Time 0 and Time 4 (by factors of 5–19 and 10–28, respectively). One week after de-fruiting, the expression of all these genes was significantly increased, and within 2 weeks they attained their maximal levels. While the mRNA levels of CAX-like, NPH3-like, and Ca^2+^-binding EF-Hand1-like remained high 4 weeks after de-fruiting, those of PBP1-like1 and PBP1-like2 were reduced by ~2-fold and 1.5-fold, respectively, during the same period.

### Auxin levels are significantly higher in ON-Crop buds and decrease following fruit removal

**Fig. 9.**
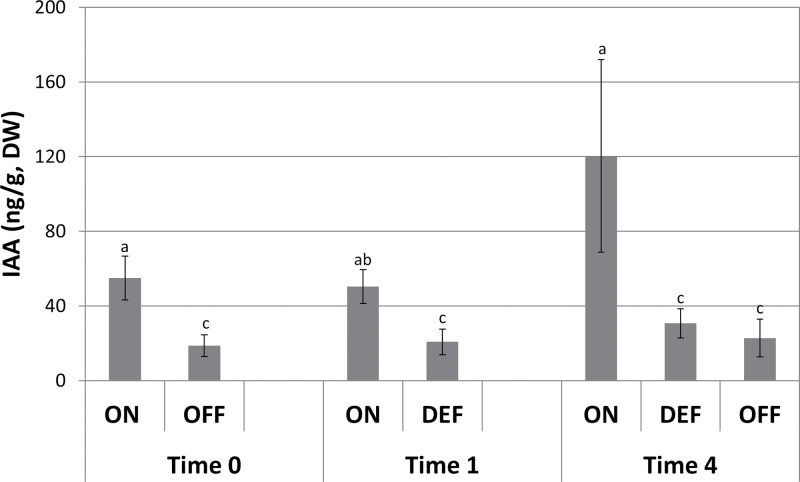
Fruit load affects auxin level in buds. Auxin was determined at the indicated time points in buds of ON-Crop (ON), OFF-Crop (OFF), and de-fruited (DEF) trees. The numbers are mean values of three independent biological replicates. Means followed by different letters are significantly different (*P*>0.05) according to Tukey–Kramer multiple comparisons tests. (This figure is available in colour at *JXB* online.)

Next, levels of endogenous IAA were examined in the buds (Fig. 9). IAA levels were significantly higher in ON-Crop buds relative to OFF-Crop buds by 2.9-fold at Time 0 and by 5.2-fold at Time 4. As expected, IAA levels decreased in DEF buds relative to ON-Crop buds, by a factor of 2.4 at Time 1, and remained at this level 4 weeks after de-fruiting.

## Discussion

### Intensity of return bloom is affected by de-fruiting, which induces relatively rapid changes in expression of flowering control genes and in the transcriptome

Fruit removal has been reported to be effective in inducing return bloom (Monselise and Goldschmidt, 1981). However, since annual variation and cultivar-dependent divergence may affect its effectiveness (Verreynne and Lovatt, 2009; Martinez-Fuentes *et al.*, 2010; Muñoz-Fambuena *et al.*, 2011), fruit removal was carried out as early as the last 10 d of August. Indeed, the number of inflorescences and vegetative shoots, counted during the following spring (Fig. 1), showed that those of DEF trees were similar to those of OFF-Crop trees, thus demonstrating the effectiveness of the treatment. Although it could be expected that the number of generative inflorescences would be higher in DEF than OFF-Crop trees (Verreynne and Lovatt, 2009), the actual number was identical, probably due to the low number of fruits on OFF-Crop trees (~45-fold lower than in ON-Crop trees). De-fruiting resulted in relatively rapid changes in the expression of genes controlling and genes associated with trehalose and flavonoid biosynthesis, allowing determination of the time frame of the genomic analysis.

As a rule, the expression of flowering control genes is induced in leaves, buds, and stems in association with the onset of the flowering induction period (November–December) in regular bearer cultivars, and in AB cultivars during the OFF-Crop year, while GA treatment reduced their expression (Nishikawa *et al.*, 2007; Muñoz-Fambuena *et al.*, 2011, 2012*b*; Shalom *et al.*, 2012; Goldberg-Moeller *et al.*, 2013). *CiSPL5* is an exception to this rule, probably due to its highly regulatory role, and it exhibited higher expression in buds from May until September (Shalom *et al.*, 2012). *CiFT2* expression was usually earlier than the onset of the flowering induction period (Shalom *et al.*, 2012). Taking into account these expression patterns and the expected year to year alternation, the mRNA levels of *CiFT2* and *CiSPL5* were higher in OFF- than in ON-Crop buds. Therefore, the increase in the expression of *CiFT2* and *CiSPL5* in buds of DEF trees to levels similar to those in buds of OFF-Crop trees can be expected, and reinforces their role in return bloom. It is not surprising that during the time of the experiment, *LFY* did not show any difference in its expression between buds of ON- and OFF-Crop trees. Nevertheless, de-fruiting resulted in a 2-fold increase in its mRNA level, which returned to its basal level 4 weeks after treatment. Whether this temporary response has any relationship to return bloom requires further research.

The differences between bud populations—those with a 55% chance to flower (ON-Crop) and those with a 96% chance (OFF-Crop and DEF buds)—did not seem to be very high. However, genomic analysis resulted in numerous differentially expressed genes (DEGs), allowing the partial identification of mechanisms that convert ON into OFF buds. Previously it was shown that the number of DEGs between ON- and OFF-Crop buds was considerably lower in September than in May (Shalom *et al.*, 2012). However, the present work showed that the number of these DEGs was quite high. This difference could be explained by year to year alternations, and differences in the methodologies used. Based on a cut-off of 50% coverage between sequences on the microarray and the currently identified sequences, and on at least 75% identity, it is estimated that only ~30–35% of the current sequences are present on the microarray, supporting this notion. Only 40% of the auxin transport-related genes (Fig. 8A) were found on the microarray. Below, three identified mechanisms, common to OFF and DEF buds, which are altered during the conversion of DEF buds into OFF buds, and might play a role in the signalling mechanism of fruit load are discussed.

### Induction of photosynthetic gene expression and protein levels in the bud following de-fruiting

In agreement with Shalom *et al.* (2012), this study demonstrates that de-fruiting induces expression of photosynthetic genes in the bud. Although a recent proteomic analysis did not show an increase in photosynthetic proteins in OFF-Crop trees (Muñoz-Fambuena *et al.*, 2013), here the induced gene expression resulted in increased protein levels of four major genes. According to the C/N theory, the proteins of photosynthetic machinery represent the majority of leaf nitrogen which is directly related to photosynthetic capacity (Evans, 1989); thus, the induced levels of photosynthesis proteins would suggest the induction of photosynthesis in OFF buds, although direct evidence is missing. Although bud photosynthesis was never measured in fruit trees, leaf photosynthesis in relation to fruit load has been measured in previous studies. While some workers found no change in photosynthesis between leaves of ON- and OFF-Crop trees (Roper *et al.*, 1988; Monerri *et al.*, 2011; Nebauer *et al.*, 2013), others reported increased photosynthetic and CO_2_ assimilation rates in fruit-bearing as compared with non-fruit-bearing trees (Fujii and Kennedy, 1985; Dejong, 1986; Gucci *et al.*, 1995; Palmer *et al.*, 1997; Iglesias *et al.*, 2002; Syvertsen *et al.*, 2003; Urban *et al.*, 2004). Vegetative growth is induced in buds of OFF-Crop and DEF trees (Monselise and Goldschmidt, 1982), suggesting that increased photosynthesis may mark the initiation of vegetative growth. That is, due to fruit absence, the OFF-Crop and DEF trees are heavily loaded with photoassimilates, suggesting that by induction of its photosynthetic machinery, the bud signals to stop translocation of photoassimilates. The possibility that the flow of photoassimilates into the bud is reduced due to lower leaf photosynthesis in OFF-Crop trees, resulting in increased synthesis of photosynthesis proteins and higher CO_2_ assimilation, cannot be excluded.

### Bud ABA is reduced in OFF-Crop trees and following de-fruiting compared with ON-Crop trees

Increased expression of three *NCED-like* genes, in buds of DEF and OFF-Crop trees compared with buds of ON-Crop trees, suggests the induction of ABA biosynthesis. However, direct measurements of ABA and its catabolites showed the opposite trend, namely reduced levels in buds of OFF-Crop and DEF trees. Direct biochemical evidence demonstrated that *NCED3* (*Cs5g14370*) cleaved 9-*cis*-violaxanthin to form xanthoxin, a precursor of ABA (Kato *et al.*, 2006); its expression paralleled ABA levels in the peel and during cycles of drought and re-watering of leaves and fruit (Rodrigo *et al.*, 2006; Agustí *et al.*, 2007). Therefore, one would expect higher ABA levels in OFF-Crop and DEF buds than in ON-Crop buds. A possible explanation of these apparently contradictory results is that the source of ABA in the ON-Crop bud is not within the bud itself, but external to it, and dependent on the presence of fruit. In OFF-Crop trees or following de-fruiting, the translocation of ABA from this source into the bud is blocked, at least partially, reducing the bud’s ABA contents, and inducing *NCDE3* expression in order to increase endogenous ABA production. Nevertheless, the possibility cannot be excluded that induced expression of *NCED* genes is futile, and has no physiological role. A closer look at ABA-responsive genes in the transcriptomic data (overall, eight differentially expressed genes between the states) did not solve this contradiction, as no common trend in their response was evident (data not shown). Regardless, ABA levels (and the expression of ABA receptor component, *PYR1-like*) in OFF-Crop buds and following de-fruiting were reduced, raising the question of its possible involvement in AB control. Consistent with the present results, buds of ON-Crop trees have been shown to contain higher levels of ABA or its isomer, t-ABA, than those of OFF-Crop trees (Jones *et al.*, 1976; Goldschmidt, 1984). It has been suggested that elevated levels of ABA in ON organs may reflect a stress imposed by the fruit overload. Moreover, ABA might serve as an inhibitor of return bloom, since the local application of ABA to *Citrus unshiu* buds in late December inhibited bud sprouting and intensive flowering (Garcialuis *et al.*, 1986). Alternatively, the possibility that flowering promotes ABA activity has been suggested, since increased ABA levels were detected in leaves of OFF-Crop trees and following de-fruiting of ON-Crop trees in association with flowering induction (Koshita *et al.*, 1999; Okuda, 2000). Whether ABA plays a role in AB control, or in other processes, such as maintaining the bud in an inactive state (Little and Edit, 1968; Horvath *et al.*, 2003; Shalom *et al.*, 2012), requires further investigation.

### De-fruiting induces genes of calcium-dependent auxin polar transport

The results showed an increase in the expression of calcium-related genes together with significant reduction in auxin levels in OFF-Crop buds and in buds following de-fruiting as compared with ON-Crop buds. Changes in the concentration of cytosolic free Ca^2+^ ([Ca^2+^]_cyt_), mediated by ion channels, Ca^2+^-ATPases, and Ca^2+^/H^+^ antiporters, form the basis of the Ca^2+^ signalling mechanism. The CAX-type antiporters are a family of cytosolic low-affinity Ca^2+^/H^+^ antiporters, which in *Arabidopsis* comprises six members. In *Citrus* there are four highly CAX homologous genes, and the expression of a CAX3 homologue was highly induced following de-fruiting. Transduction of Ca^2+^ signals is carried out by specific calcium-binding proteins, containing a common structural motif called the ‘EF-hand’, a helix–loop–helix structure that binds a single Ca^2+^ ion (Day *et al.*, 2002). The present results showed a significant up-regulation of a few genes encoding EF-hand proteins in OFF and DEF buds compared with their level in ON buds. Overall, these results might suggest that [Ca^2+^]_cyt_ is affected by fruit load, although at this stage a plausible scenario as to the nature of the change and its cellular signature cannot be suggested. How are these changes related to auxin polar transport? Four of the up-regulated EF-hand genes show remarkable homology to the genes encoding PBP1 in *Arabidopsis*. PBP1 interacts physically with PID protein kinase, regulating its activity in response to changes in calcium levels (Benjamins *et al.*, 2003). PID regulates the polarity of PIN proteins (Friml *et al.*, 2004), which are known to direct auxin flow (Wisniewska *et al.*, 2006). NPH3-like proteins have recently been shown to affect PIN localization (Furutani *et al.*, 2011; Wan *et al.*, 2012). As shown here, *NPH3-like* genes are part of a relatively large gene family. Divergence of its different members occurred before the divergence of *Arabidopsis* and *Citrus*. The citrus *NPH3-like* gene induced in OFF and DEF buds compared with ON buds showed very close homology to an *Arabidopsis* gene which has not yet been subjected to detailed analysis.

Taken together, the present results lead to the suggestion that higher levels of IAA in ON buds reflect their inability to distribute IAA efficiently via the Ca^2+^-dependent PIN-based polar auxin transport mechanism. In addition, efficient auxin removal from the bud appears to be a key component in transforming the ON bud into an OFF bud. The involvement of auxin in flowering inhibition following an ON-Crop year was recently suggested (Smith and Samach, 2013), and is based on the ATA hypothesis proposed by Bangerth (Bangerth, 1989, 2006; Caaejas and Bangerth, 1997). The application of auxin polar transport inhibitors resulted in flowering induction in a number of fruit trees (Bukovac, 1968; Ben-Tal and Lavee, 1985; Ito *et al.*, 2001; Blaikie *et al.*, 2004; Bangerth, 2006). The strong polar transport of auxin from the dominant sinks (i.e. the fruit or the seed), as suggested by the ATA hypothesis, preventing auxin export from the bud, would explain why auxin levels in OFF buds and in buds following de-fruiting are lower than in ON buds.

The parallel reduction in ABA and IAA levels in the bud would suggest cross-talk between the ABA and IAA signalling pathways. Such cross-talk interactions were suggested in *Arabidopsis* embryo axis elongation and root development (Belin *et al.*, 2009; Shkolnik-Inbar and Bar-Zvi, 2010; Wang *et al.*, 2011), but not in flowering control processes.

## Supplementary data

Supplementary data are available at *JXB* online.

Supplementary File 1

Figure S1. De-fruiting alters the expression of trehalose- and flavonoid-metabolizing genes.

Figure S2. Venn diagrams of developmentally regulated genes.

Figure S3. Photosynthetic genes are induced following de-fruiting in the bud.

Figure S4. Quantification of protein blot results (Fig. 5C).

Figure S5. Schematic representation of the cleavage of 9-*cis* xanthophylls to xanthoxin by 9-*cis*-epoxycarotenoid dioxygenase (NCED), a key regulated step in the biosynthesis of ABA in plants.

Figure S6. Changes in the expression of the *PYR1-like* gene.

Figure S7. Linear regression between transcriptomic and transcriptional (qPCR) data of Ca-related and *NPH3-like* genes, presented in Fig. 8A and B.

Figure S8. Genes encoding PINOID (PID)-binding protein 1 (PBP1) and Non-Phototropic Hypocotyl 3-like (NPH3) show homology in *Arabidopsis* and *Citrus*.

Table S1. List of primers used in this study.

Table S2. List of genes used to design probes for nCounter analysis (Shalom *et al.*, 2012).

Table S3. Statistical data of deep sequencing analysis.

Table S4. GO categorization of unique genes up- or down-regulated in buds of OFF-Crop and de-fruited (DEF) trees relative to buds of ON-Crop trees during Time 4.

Table S5. GO categorization of genes of cluster 4 of DEF buds.

Table S6. GO categorization of up-regulated genes of OFF-Crop buds.

Table S7. GO categorization of down-regulated genes of OFF-Crop buds.

Table S8. GO categorization of genes of cluster 3 of DEF buds.

Table S9. GO categorization of genes of cluster 2 of ON-Crop buds.

Supplementary File 2

The file includes: (i) all possible comparisons between the treatments; (ii) the accession numbers of genes presented in the Venn diagrams (Fig. 4A, B; Supplementary S2A, B); (iii) photosynthesis genes (Fig. 5A); and (iv) calcium-related genes (Fig. 8A).

Supplementary Data

## References

[CIT0001] Agustí J Zapater M Iglesias DJ Cercos M Tadeo FR Tálon M 2007 Differential expression of putative 9-cis-epoxycarotenoid dioxygenases and abscisic acid accumulation in water stressed vegetative and reproductive tissues of citrus. Plant Science 172, 85–94

[CIT0002] Albrigo LG Galán-Saúco V 2004 Flower bud induction, flowering and fruit-set of some tropical and subtropical fruit tree crops with special reference to citrus. Acta Horticulturae 632, 81–91

[CIT0003] Bangerth F 1989 Dominance among fruits sinks and the search for a correlative signal. Physiologia Plantarum 76, 608–614

[CIT0004] Bangerth F 2006 Flower induction in perennial fruit trees: still an enigma? Acta Horticulturae 727, 177–195

[CIT0005] Bangerth KF 2009 Floral induction in mature, perennial angiosperm fruit trees: milarities and discrepancies with annual/biennial plants and the involvement of plant hormones. Scientia Horticulturae 122, 153–163

[CIT0006] Belin C Megies C Hauserova E Lopez-Molina L 2009 Abscisic acid represses growth of the arabidopsis embryonic axis after germination by enhancing auxin signaling. The Plant Cell 21, 2253–2268 19666738 10.1105/tpc.109.067702 PMC2751952

[CIT0007] Ben-Tal Y Lavee S 1985 Girdling olive trees, a partial solution to biennial bearing. III. Chemical girdling: its influence on flowering and yield. Rivista della Ortoflorofrutticoltura Italiana 69, 1–11

[CIT0008] Benjamins R Ampudia CSG Hooykaas PJJ Offringa R 2003 PINOID-mediated signaling involves calcium-binding proteins. Plant Physiology 132, 1623–1630 12857841 10.1104/pp.103.019943 PMC167099

[CIT0009] Blaikie SJ Kulkarni VJ Muller WJ 2004 Effects of morphactin and paclobutrazol flowering treatments on shoot and root phenology in mango cv. Kensington Pride. Scientia Horticulturae 101, 51–68

[CIT0010] Bower JP Lovatt CJ Cutting JGM Blanke MM 1990 Interaction of plant growth regulators and carbohydrate in flowering and fruit set. Acta Horticulturae 275, 425–434

[CIT0011] Bukovac MJ 1968 TIBA promotes flowering and wide branch angles. American Fruit Grower 88, 18

[CIT0012] Caaejas R Bangerth F 1997 Is auxin export of apple fruit an alternate signal for inhibition of flowering bud induction. Acta Horticulturae 463, 271–277

[CIT0013] Day IS Reddy VS Ali GS Reddy ASN 2002 Analysis of EF-hand-containing proteins in Arabidopsis. Genome Biology 3, RESEARCH0056 12372144 10.1186/gb-2002-3-10-research0056 PMC134623

[CIT0014] Dejong TM 1986 Fruit effects on photosynthesis in Prunus persica. Physiologia Plantarum 66, 49–153

[CIT0015] Dennis FG 2000 The history of fruit thinning. Plant Growth Regulation 31, 1–16

[CIT0016] Eriksson S Böhlenius H Moritz T Nilsson O 2006 GA4 is the active gibberellin in the regulation of LEAFY transcription and Arabidopsis floral initiation. The Plant Cell 18, 2172–2181 16920780 10.1105/tpc.106.042317 PMC1560906

[CIT0017] Evans JR 1989 Photosynthesis and nitrogen relationships in leaves of C-3 plants. Oecologia 78, 9–19 10.1007/BF00377192 28311896

[CIT0018] Friml J Yang X Michniewicz M 2004 A PINOID-dependent binary switch in apical–basal PIN polar targeting directs auxin efflux. Science 306, 862–865 15514156 10.1126/science.1100618

[CIT0019] Fujii JA Kennedy RA 1985 Seasonal changes in the photosynthetic rate in apple trees—a comparison between fruiting and nonfruiting trees. Plant Physiology 78, 519–524 16664276 10.1104/pp.78.3.519 PMC1064769

[CIT0020] Furutani M Sakamoto N Yoshida S Kajiwara T Robert HS Friml J Tasaka M 2011 Polar-localized NPH3-like proteins regulate polarity and endocytosis of PIN-FORMED auxin efflux carriers. Development 138, 2069–2078 21490067 10.1242/dev.057745

[CIT0021] Garcialuis A Almela V Monerri C Agustí M Guardiola JL 1986 Inhibition of flowering in vivo by existing fruits and applied growth-regulators in Citrus unshiu. Physiologia Plantarum 66, 515–520

[CIT0022] Goldberg-Moeller R Shalom L Shlizerman L Samuels S Zur N Ophir R Blumwald E Sadka A 2013 Effects of gibberellin treatment during flowering induction period on global gene expression and the transcription of flowering-control genes in Citrus buds. Plant Science 198, 46–57 23199686 10.1016/j.plantsci.2012.09.012

[CIT0023] Goldschmidt EE 1984 Endogenous abscisic acid and 2-*trans*-abscisic acid in alternate bearing ‘Wilking’ mandarin trees. Plant Growth Regulation 2, 9–13

[CIT0024] Goldschmidt EE 1999 Carbohydrate supply as a critical factor for citrus fruit development and productivity. HortScience 34, 1020–1024

[CIT0025] Goldschmidt EE 2013 The evolution of fruit tree productivity: a review. Economic Botany 67, 51–62 23538880 10.1007/s12231-012-9219-y PMC3606516

[CIT0026] Goldschmidt EE Aschkenazi N Herzano Y Schaffer AA Monselise SP 1985 A role for carbohydrate-levels in the control of flowering in citrus. Scientia Horticulturae 26, 159–166

[CIT0027] Goldschmidt EE Golomb A 1982 The carbohydrate balance of alternate-bearing citrus trees and the significance of reserves for flowering and fruiting. Journal of the American Society for Horticultural Science 107, 206–208

[CIT0028] Goldschmidt EE Samach A 2004 Aspects of flowering in fruit trees. Acta Horticulturae 653, 23–27

[CIT0029] Goldschmidt EE Tamim M Goren R 1997 Gibberellins and flowering in Citrus and other fruit trees: a critical analysis. Acta Horticulturae 463, 201–208

[CIT0030] Gucci R Grappadelli LC Tustin S Ravaglia G 1995 The effect of defruiting at different stages of fruit development on leaf photosynthesis of Golden Delicious apple. Tree Physiology 15, 35–40 14966009 10.1093/treephys/15.1.35

[CIT0031] Hilgeman RH Dunlap JA Sharpies GC 1967 Effect of time of harvest of Valencia oranges on leaf carbohydrate content and subsequent set of fruit. Proceedings of the American Society for Horticultural Science 90, 110–116

[CIT0032] Horvath DP Anderson JV Chao WS Foley ME 2003 Knowing when to grow: signals regulating bud dormancy. Trends in Plant Science 8, 534–540 14607098 10.1016/j.tplants.2003.09.013

[CIT0033] Iglesias DJ Lliso I Tadeo FR Tálon M 2002 Regulation of photosynthesis through source:sink imbalance in citrus is mediated by carbohydrate content in leaves. Physiologia Plantarum 116, 563–572

[CIT0034] Ito A Hayama H Kaskimura Y Yoshioka H 2001 Effect of maleic hydrazide on endogenous cytokinin contents in lateral buds, and its possible role in flower bud formation on the Japanese pear shoot. Scientia Horticulturae 87, 199–205

[CIT0035] Jones WW Coggins CW Embleton TW 1976 Endogenous abscisic acid in relation to bud growth in alternate bearing Valencia orange. Plant Physiology 58, 681–682 16659743 10.1104/pp.58.5.681 PMC542282

[CIT0036] Jones WW Embleton TW Barnhart EL Cree CB 1974 Effect of time and amount of fruit thinning on leaf carbohydrate and fruit set in ‘Valencia’ oranges. Hilgardia 42, 441–449

[CIT0037] Jones WW Embleton TW Steinacker ML Cree CB 1970 Carbohydrates and fruiting of Valencia oranges trees. Journal of the American Society for Horticultural Science 95, 380–381

[CIT0038] Kato M Matsumoto H Ikoma Y Okuda H Yano M 2006 The role of carotenoid cleavage dioxygenases in the regulation of carotenoid profiles during maturation in citrus fruit. Journal of Experimental Botany 57, 2153–2164 16714310 10.1093/jxb/erj172

[CIT0039] Kim D Pertea G Trapnell C Pimentel H Kelley R Salzberg SL 2013 TopHat2: accurate alignment of transcriptomes in the presence of insertions, deletions and gene fusions. Genome Biology 14, R36 23618408 10.1186/gb-2013-14-4-r36 PMC4053844

[CIT0040] Knauer T Dummer M Landgraf F Forreiter C 2011 A negative effector of blue light-induced and gravitropic bending in Arabidopsis. Plant Physiology 156, 439–447 21367967 10.1104/pp.110.167411 PMC3091041

[CIT0041] Koshita Y Takahara T Ogata T Goto A 1999 Involvement of endogenous plant hormones (IAA, ABA, GAs) in leaves and flower bud formation of satsuma mandarin (Citrus unshiu Marc.). Scientia Horticulturae 79, 185–194

[CIT0042] Kotoda N Hayashi H Suzuki M 2010 Molecular characterization of FLOWERING LOCUS T-like genes of apple (Malus×domestica Borkh.). Plant and Cell Physiology 51, 561–575 20189942 10.1093/pcp/pcq021

[CIT0043] Li CY Weiss D Goldschmidt EE 2003a Effects of carbohydrate starvation on gene expression in citrus root. Planta 217, 11–20 12721844 10.1007/s00425-002-0963-6

[CIT0044] Li CY Weiss D Goldschmidt EE 2003b Girdling affects carbohydrate-related gene expression in leaves, bark and roots of alternate-bearing citrus trees. Annals of Botany 92, 137–143 12763756 10.1093/aob/mcg108 PMC4243633

[CIT0045] Li YT Dai XH Cheng YF Zhao YD 2011 NPY genes play an essential role in root gravitropic responses in Arabidopsis. Molecular Plant 4, 171–179 20833732 10.1093/mp/ssq052 PMC3026118

[CIT0046] Little CHA Edit DC 1968 Effect of abscisic acid on budbreak and transpiration in woody species. Nature 220, 498–499

[CIT0047] Lohse M Nagel A Herter T May P Schroda M Zrenner R Tohge T Fernie AR Stitt M Usadel B 2013 Mercator: a fast and simple web server for genome scale functional annotation of plant sequence data. Plant, Cell and Environment (in press). 10.1111/pce.12231 24237261

[CIT0048] Maayan I Shaya F Ratner K Mani Y Lavee S Avidan B Shahak Y Ostersetzer-Biran O 2008 Photosynthetic activity during olive (Olea europaea) leaf development correlates with plastid biogenesis and Rubisco levels. Physiologia Plantarum 134, 547–558 18636989 10.1111/j.1399-3054.2008.01150.x

[CIT0049] Martinez-Fuentes A Mesejo C Reig C Agustí M 2010 Timing of the inhibitory effect of fruit on return bloom of ‘Valencia’ sweet orange (Citrus sinensis (L.) Osbeck). Journal of the Science of Food and Agriculture 90, 1936–1943 20564309 10.1002/jsfa.4038

[CIT0050] Monerri C Fortunato-Almeida A Molina RV Nebauer SG Garcia-Luis A Guardiola JL 2011 Relation of carbohydrate reserves with the forthcoming crop, flower formation and photosynthetic rate, in the alternate bearing ‘Salustiana’ sweet orange (Citrus sinensis L.). Scientia Horticulturae 129, 71–78

[CIT0051] Monselise SP Goldschmidt EE 1981 Alternate bearing in citrus and ways of control. Proceedings of the International Society of Citriculture 1, 239–242

[CIT0052] Monselise SP Goldschmidt EE 1982 Alternate bearing in fruit trees. Horticultural Reviews 4, 128–173

[CIT0053] Muñoz-Fambuena N Mesejo C Agustí M Tarraga S Iglesias DJ Primo-Millo E González-Mas MC 2013 Proteomic analysis of ‘Moncada’ mandarin leaves with contrasting fruit load. Plant Physiology and Biochemistry 62, 95–106 23202483 10.1016/j.plaphy.2012.10.020

[CIT0054] Muñoz-Fambuena N Mesejo C González-Mas MC Iglesias DJ Primo-Millo E Agustí M 2012a Gibberellic acid reduces flowering intensity in sweet orange [Citrus sinensis (L.) Osbeck] by repressing CiFT gene expression. Journal of Plant Growth Regulation 31, 529–536

[CIT0055] Muñoz-Fambuena N Mesejo C González-Mas MC Primo-Millo E Agustí M Iglesias DJ 2011 Fruit regulates seasonal expression of flowering genes in alternate-bearing ‘Moncada’ mandarin. Annals of Botany 108, 511–519 21856639 10.1093/aob/mcr164 PMC3158683

[CIT0056] Muñoz-Fambuena N Mesejo C González-Mas MC Primo-Millo E Agustí M Iglesias DJ 2012b Fruit load modulates flowering-related gene expression in buds of alternate-bearing ‘Moncada’ mandarin. Annals of Botany 110, 1109–1118 22915579 10.1093/aob/mcs190 PMC3478051

[CIT0057] Nakagawa M Honsho C Kanzaki S Shimizu K Utsunomiya N 2012 Isolation and expression analysis of FLOWERING LOCUS T-like and gibberellin metabolism genes in biennial-bearing mango trees. Scientia Horticulturae 139, 108–117

[CIT0058] Nebauer SG Arenas C Rodriguez-Gamir J Bordon Y Fortunato-Almeida A Monerri C Guardiola JL Molina RV 2013 Crop load does not increase the photosynthetic rate in Citrus leaves under regular cropping conditions. A study throughout the year. Scientia Horticulturae 160, 358–365

[CIT0059] Nishikawa F Endo T Shimada T Fujii H Shimizu T Omura M Ikoma Y 2007 Increased CiFT abundance in the stem correlates with floral induction by low temperature in Satsuma mandarin (*Citrus unshiu* Marc.). Journal of Experimental Botany 58, 3915–3927 18000016 10.1093/jxb/erm246

[CIT0060] Okuda H 2000 A comparison of IAA and ABA levels in leaves and roots of two citrus cultivars with different degrees of alternate bearing. Journal of Horticultural Science and Biotechnology 75, 355–359

[CIT0061] Palmer JW Giuliani R Adams HM 1997 Effect of crop load on fruiting and leaf photosynthesis of ‘Braeburn’/M.26 apple trees. Tree Physiology 17, 741–746 14759899 10.1093/treephys/17.11.741

[CIT0062] Rodrigo MJ Alquezar B Zacarias L 2006 Cloning and characterization of two 9-*cis*-epoxycarotenoid dioxygenase genes, differentially regulated during fruit maturation and under stress conditions, from orange (*Citrus sinensis* L. Osbeck). Journal of Experimental Botany 57, 633–643 16396998 10.1093/jxb/erj048

[CIT0063] Roper TR Keller JD Loescher WH Rom CR 1988 Photosynthesis and carbohydrate partitioning in sweet cherry—fruiting effects. Physiologia Plantarum 72, 42–47

[CIT0064] Shalom L Samuels S Zur N Shlizerman L Zemach H Weissberg M Ophir R Blumwald E Sadka A 2012 Alternate bearing in citrus: changes in the expression of flowering control genes and in global gene expression in ON- versus OFF-Crop trees. PLoS One 7, e46930 23071667 10.1371/journal.pone.0046930 PMC3469648

[CIT0065] Shkolnik-Inbar D Bar-Zvi D 2010 ABI4 mediates abscisic acid and cytokinin inhibition of lateral root formation by reducing polar auxin transport in Arabidopsis. The Plant Cell 22, 3560–3573 21097710 10.1105/tpc.110.074641 PMC3015119

[CIT0066] Smith HM Samach A 2013 Constraints to obtaining consistent annual yields in perennial tree crops. I: heavy fruit load dominates over vegetative growth. Plant Science 207, 158–167 23602111 10.1016/j.plantsci.2013.02.014

[CIT0067] Suzek BE Huang HZ McGarvey P Mazumder R Wu CH 2007 UniRef: comprehensive and non-redundant UniProt reference clusters. Bioinformatics 23, 1282–1288 17379688 10.1093/bioinformatics/btm098

[CIT0068] Syvertsen JP Goni C Otero A 2003 Fruit load and canopy shading affect leaf characteristics and net gas exchange of ‘Spring’ navel orange trees. Tree Physiology 23, 899–906 14532013 10.1093/treephys/23.13.899

[CIT0069] Tálon M Tadeo FR Ben-Cheikh W Gomez-Cadenas A Mehouachi J Pérez-Botella J Primo-Millo E 1997 Hormonal regulation of fruit set and abscission in citrus: classical concepts and new evidence. Acta Horticulturae 463, 209–217

[CIT0070] Tan BC Joseph LM Deng WT Liu LJ Li QB Cline K McCarty DR 2003 Molecular characterization of the Arabidopsis 9-cis epoxycarotenoid dioxygenase gene family. The Plant Journal 35, 44–56 12834401 10.1046/j.1365-313x.2003.01786.x

[CIT0071] Trapnell C Williams BA Pertea G Mortazavi A Kwan G van Baren MJ Salzberg SL Wold BJ Pachter L 2010 Transcript assembly and quantification by RNA-Seq reveals unannotated transcripts and isoform switching during cell differentiation. Nature Biotechnology 28, 511–515 10.1038/nbt.1621 PMC3146043 20436464

[CIT0072] Urban L Lechaudel M Lu P 2004 Effect of fruit load and girdling on leaf photosynthesis in *Mangifera indica* L. Journal of Experimental Botany 55, 2075–2085 15310823 10.1093/jxb/erh220

[CIT0073] Usadel B Poree F Nagel A Lohse M Czedik-Eysenberg A Stitt M 2009 A guide to using MapMan to visualize and compare omics data in plants: a case study in the crop species, maize. Plant, Cell and Environment 32, 1211–1229 10.1111/j.1365-3040.2009.01978.x 19389052

[CIT0074] Valiente JI Albrigo LG 2004 Flower bud induction of sweet orange trees [Citrus sinensis (L.) Osbeck]: effect of low temperatures, crop load, and bud age. Journal of the American Society for Horticultural Science 129, 158–164

[CIT0075] van Dijken AJH Schluepmann H Smeekens SCM 2004 Arabidopsis trehalose-6-phosphate synthase 1 is essential for normal vegetative growth and transition to flowering. Plant Physiology 135, 969–977 15181208 10.1104/pp.104.039743 PMC514131

[CIT0076] Verreynne JS Lovatt CJ 2009 The effect of crop load on budbreak influences return bloom in alternate bearing ‘Pixie’ mandarin. Journal of the American Society for Horticultural Science 134, 299–307

[CIT0077] Wahl V Ponnu J Schlereth A Arrivault S Langenecker T Franke A Feil R Lunn JE Stitt M Schmid M 2013 Regulation of flowering by trehalose-6-phosphate signaling in Arabidopsis thaliana. Science 339, 704–707 23393265 10.1126/science.1230406

[CIT0078] Wan YL Jasik J Wang L Hao HQ Volkmann D Menzel D Mancuso S Baluska F Lin JX 2012 The signal transducer NPH3 integrates the phototropin1 photosensor with PIN2-based polar auxin transport in Arabidopsis root phototropism. The Plant Cell 24, 551–565 22374399 10.1105/tpc.111.094284 PMC3315232

[CIT0079] Wang L Hua DP He JN Duan Y Chen ZZ Hong XH Gong ZZ 2011 Auxin response factor2 (ARF2) and its regulated homeodomain gene HB33 mediate abscisic acid response in Arabidopsis. PLoS Genetics 7, e1002172 21779177 10.1371/journal.pgen.1002172 PMC3136439

[CIT0080] Wisniewska J Xu J Seifertova D Brewer PB Ruzicka K Blilou I Rouquie D Benkova E Scheres B Friml J 2006 Polar PIN localization directs auxin flow in plants. Science 312, 883–883 16601151 10.1126/science.1121356

[CIT0081] Xu Q Chen LL Ruan XA 2013 The draft genome of sweet orange (Citrus sinensis). Nature Genetics 45, 59–66 23179022 10.1038/ng.2472

