# Supplementary material for: Fruit load induces changes in global gene expression and in abscisic acid (ABA) and indole acetic acid (IAA) homeostasis in citrus buds
Source: J Exp Bot. 2014 Apr 4;65(12):3029–44. doi: 10.1093/jxb/eru148 (PMC4071824; doi:10.1093/jxb/eru148)
Supplement: Supplementary Data [file supp_65_12_3029__index.html]

Fruit load induces changes in global gene expression and in abscisic acid (ABA) and indole acetic acid (IAA) homeostasis in citrus buds — Fruit load induces changes in global gene expression and in abscisic acid (ABA) and indole acetic acid (IAA) homeostasis in citrus buds — Supplementary Data 

# Fruit load induces changes in global gene expression and in abscisic acid (ABA) and indole acetic acid (IAA) homeostasis in citrus buds

## Supplementary Data

Data files

**Files in this Data Supplement:**

- Supplementary Data - Supplementary Data
- Supplementary Data - Supplementary Data
