# Supplementary material for: Fruit load induces changes in global gene expression and in abscisic acid (ABA) and indole acetic acid (IAA) homeostasis in citrus buds
Source: J Exp Bot. 2014 Apr 4;65(12):3029–44. doi: 10.1093/jxb/eru148 (PMC4071824; doi:10.1093/jxb/eru148)
Supplement: Supplementary Data [file supp_eru148_jexbot114504_file002.pdf]

Fruit Load Induces Changes In Global Gene Expression And In ABA And IAA Homeostasis in Citrus Buds. *Liron Shalom, Sivan Samuels, Naftali Zur, Lyudmila Shlizerman, Adi Faigenboim, Eduardo Blumwald and Avi Sadka*

SUPPLEMENTARY DATA

Table S1. List of primers used in this study

gene name	primers sequences	
β ACTIN	GTCTGGTCCATCCATTGTCCA	Forward Primer
	CAATGGCCCCAACCTTAGC	Reverse Primer
CiFT2	CAACAAAATTTCATCACTTGAATAGTC	Forward Primer
	AAACACTCAACAACACTTAGCACAAA	Reverse Primer
	AGGCTGTGTGTGCGTGTA	dual-labeled probe
CiLFY	AATGCCCAACCAAGGTGACTA	Forward Primer
	AAAGCCCTCCTCAGTGCATTC	Reverse Primer
CiSPL5	CCTGTTGTGATTGTTGCTGGTC	Forward Primer
	CTTCCGACGCCTCTCATTGT	Reverse Primer
RbcS	CACCAACTGGCTTGAAGAAG	Forward Primer
	CACCCATCCTTTCTCCAACT	Reverse Primer
LHCB3	CCAAGTACACAATGGGAAATGA	Forward Primer
	ATCAGCAGACAAACCAGCAG	Reverse Primer
PRK	GCCATGAGGTCTCGATTTTG	Forward Primer
	CTTCAACATTTGCTGGGTGA	Reverse Primer
PSB28	TTGCTGCTGCTACTGATGTTC	Forward Primer
	CACGGATTCCTCTCTTCTCAG	Reverse Primer
PSAD	CAAACAAAGGAGATTGGCTTC	Forward Primer
	ACCAGTACCTGAATCCTGAGC	Reverse Primer
SHM	CGCCATGATGTTGAGGAGTAT	Forward Primer
	CCTCGTCGCCTCTGTGTAATA	Reverse Primer
Fd	CTGGGGGAGAGGAAGAAATC	Forward Primer
	GGTCAACGGTTCCAGACACA	Reverse Primer
NCED3-like	TGGCTACATTCTCGCTTTTG	Forward Primer
	GCTCCAGAGTCATTGCGTTA	Reverse Primer
CAX3-like	CAGAGCACTGCGAACATGG	Forward Primer
	TCAATGTACCACAGGGTCCTC	Reverse Primer
PBP1-like1	GATTGTACTCAGTCGTCCATTCC	Forward Primer
	CTCTCTCATTGTCTGGTTTTTGG	Reverse Primer
NPH3-like	TGGAAAATGAACTGGATGGA	Forward Primer
	CCTCTTTCACCTCCGACAAT	Reverse Primer
PBP1-like2	GCAAATAGTGGGGTTGTGTTT	Forward Primer
	CTCATCCATTAGCAAACGAAAC	Reverse Primer
Ca-binding EF-hand1	GCTTGGAGGAACTCAACTGG	Forward Primer
	GCAGCAGCATTGGGATTATT	Reverse Primer

Table S2. List of genes used to design probes for nCounter analysis (Shalom *et al*., 2012)

Gene	Gene name
Trehalose phosphate synthase (TPS)	Ciclev10018842
Trehalose phosphate phosphatase (TPP)	Ciclev10005151
UDP-glucose:flavonoid-3-O-glucosyltransferase (UF3GT)	Ciclev10004868
4-coumarate:coenzyme A ligase (4CL)	Ciclev10019532
Chalcone synthase (CHS)	Ciclev10015535
Chalconeisomerase (CHI)	Ciclev10032749
Cinnamate 4-hydroxylase (C4H)	Ciclev10008125
β Actin	Ciclev10025866
Polyubiquitin 2	Ciclev10013001
Cyclophilin	Ciclev10029291

Table S3. Statistical data of deep sequencing analysis

Sample ID	Avg. Yield (Mb)	Avg. # Reads	Avg. # Transcripts	GO Annotated
ON Time 0	2120.5	20,993,674	12495	9715
OFF Time 0	1,896	18,767,601	12680	9762
ON Time 1	2,246	22,239,857	12444	9688
DEFTime 1	2,103	20,820,325	12532	9750
ON Time 2	2,235	22,132,161	12349	9591
DEFTime 2	2,125	21,040,717	12412	9610
ON Time 4	2,224	22,023,764	12456	9684
DEFTime 4	2,486	24,614,145	12482	9681
OFF Time 4	3,189	31,578,496	12724	9891
**Total number of transcripts in all libraries**			**14,400**	**11,445**

Table S4. GO[1] categorization of unique genes up- or down-regulated in buds of OFF-Crop and de-fruited (DEF) trees relative to buds of ON-Crop trees during Time 4.

General process	GO term	Description	Query item	Ref item	p-value	FDR	Enrichment fold[2]
**OFF-Crop up-regulated**							
Glucan/Starch	GO:0019252	starch biosynthetic process	15	51	1.00E-06	0.00083	4.33
	GO:0005982	starch metabolic process	16	61	2.40E-06	0.00099	3.86
	GO:0009311	oligosaccharide metabolic process	16	66	7.30E-06	0.002	3.57
	GO:0006073	cellular glucan metabolic process	22	146	0.00041	0.041	2.22
	GO:0044042	glucan metabolic process	23	158	0.00051	0.045	2.14
Carbohydrate metabolism	GO:0016051	carbohydrate biosynthetic process	39	308	0.00019	0.022	1.86
	GO:0000023	maltose metabolic process	12	36	2.80E-06	0.001	4.91
	GO:0005984	disaccharide metabolic process	13	55	6.70E-05	0.011	3.48
Glucosinolate metabolism	GO:0019761	glucosinolate biosynthetic process	14	65	0.00011	0.014	3.17
	GO:0019757	glycosinolate metabolic process	14	69	0.00021	0.023	2.99
	GO:0019758	glycosinolate biosynthetic process	14	65	0.00011	0.014	3.17
	GO:0019760	glucosinolate metabolic process	14	69	0.00021	0.023	2.99
S-Glycoside metabolism	GO:0016143	S-glycoside metabolic process	14	69	0.00021	0.023	2.99
	GO:0016144	S-glycoside biosynthetic process	14	65	0.00011	0.014	3.17
	GO:0016138	glycoside biosynthetic process	14	75	0.00051	0.045	2.75
Various	GO:0030104	water homeostasis	5	8	6.90E-05	0.011	9.20
	GO:0006790	sulfur metabolic process	27	196	0.00044	0.042	2.03
**DEF up-regulated**							
Endopeptidase/ hydrlase activity	GO:0010951	negative regulation of endopeptidase activity	6	9	5.90E-06	0.001	10.13
	GO:0052548	regulation of endopeptidase activity	6	10	1.40E-05	0.0021	9.12
	GO:0051346	negative regulation of hydrolase activity	8	18	8.80E-06	0.0014	6.75
	GO:0010466	negative regulation of peptidase activity	8	18	8.80E-06	0.0014	6.75
	GO:0052547	regulation of peptidase activity	8	19	1.40E-05	0.0021	6.40
dephosphorylation	GO:0035304	regulation of protein amino acid dephosphorylation	7	20	0.0002	0.016	5.32
	GO:0035303	regulation of dephosphorylation	7	22	0.00039	0.024	4.83
Salicilic acid synthesis	GO:0009697	salicylic acid biosynthetic process	8	29	0.00044	0.027	4.19
	GO:0009696	salicylic acid metabolic process	10	42	0.00033	0.023	3.62
NAD/NADPH metabolism	GO:0006769	nicotinamide metabolic process	14	70	0.00018	0.015	3.04
	GO:0046496	nicotinamide nucleotide metabolic process	14	70	0.00018	0.015	3.04
	GO:0006739	NADP metabolic process	14	66	9.10E-05	0.0094	3.22
	GO:0006740	NADPH regeneration	14	66	9.10E-05	0.0094	3.22
Oxidative stress	GO:0006979	response to oxidative stress	30	235	0.00049	0.029	1.94
	GO:0042542	response to hydrogen peroxide	15	86	0.0005	0.03	2.65
biotic stimulus	GO:0051707	response to other organism	59	544	0.0002	0.016	1.65
	GO:0009607	response to biotic stimulus	63	621	0.00073	0.04	1.54
	GO:0009620	response to fungus	25	179	0.00036	0.023	2.12
	GO:0042742	defense response to bacterium	28	219	0.00072	0.04	1.94
abiotic stimulus	GO:0009628	response to abiotic stimulus	106	898	3.00E-08	1.20E-05	
	GO:0009651	response to salt stress	35	292	0.00059	0.034	1.82
	GO:0009266	response to temperature stimulus	36	285	0.00019	0.016	1.92
endogenous stimulus	GO:0071495	cellular response to endogenous stimulus	29	226	0.00055	0.032	1.95
	GO:0009719	response to endogenous stimulus	59	508	3.20E-05	0.0041	1.76
Various	GO:0009725	response to hormone stimulus	54	466	6.90E-05	0.0078	1.76
	GO:0042221	response to chemical stimulus	130	1345	3.40E-05	0.0042	1.47
	GO:0070887	cellular response to chemical stimulus	45	366	6.50E-05	0.0076	1.87
	GO:0006733	oxidoreduction coenzyme metabolic process	14	82	0.00095	0.049	2.59
	GO:0055114	oxidation reduction	121	1344	0.00079	0.042	1.37
	GO:0018298	protein-chromophore linkage	5	10	0.00024	0.019	7.60
	GO:0022900	electron transport chain	20	98	6.10E-06	0.001	3.10
	GO:0019362	pyridine nucleotide metabolic process	14	70	0.00018	0.015	3.04
	GO:0043603	cellular amide metabolic process	14	74	0.00032	0.023	2.87
	GO:0006091	generation of precursor metabolites and energy	43	252	1.70E-08	8.90E-06	2.59
	GO:0048878	chemical homeostasis	18	111	0.00038	0.023	2.46
	GO:0006869	lipid transport	13	66	0.00035	0.023	2.99

	GO:0009820	alkaloid metabolic process	15	71	5.50E-05	0.0065	3.21
	GO:0010033	response to organic substance	86	767	3.30E-06	0.00066	1.70
**DEF down-regulated**							
**biotic stimulus**	GO:0050832	defense response to fungus	19	123	9.90E-05	0.018	2.67
	GO:0051707	response to other organism	57	544	2.50E-05	0.0071	1.81
	GO:0009620	response to fungus	25	179	5.20E-05	0.013	2.41
	GO:0009607	response to biotic stimulus	61	621	8.40E-05	0.016	1.70
**amine/amino acid metabolism**	GO:0006575	cellular amino acid derivative metabolic process	37	285	6.20E-06	0.003	2.24
	GO:0042398	cellular amino acid derivative biosynthetic process	31	208	1.90E-06	0.0014	2.58
	GO:0006519	cellular amino acid and derivative metabolic process	55	481	2.80E-06	0.0016	1.98
	GO:0009308	amine metabolic process	39	364	0.00024	0.037	1.85
	GO:0009310	amine catabolic process	11	48	7.90E-05	0.016	3.96
**aromatic compound metabolism**	GO:0006725	cellular aromatic compound metabolic process	49	388	5.70E-07	0.00082	2.18
	GO:0019438	aromatic compound biosynthetic process	31	246	5.60E-05	0.013	2.18
	GO:0010038	response to metal ion	40	354	6.80E-05	0.015	1.95

[1]Gene ontology
[2]Enrichmnet fold was calculated based on GO-annotated genes (777, 753, 821, 662 in OFF-Crop up-regulated, DEF up-regulated, OFF-Crop down-regulated, DEF down-regulated, respectively) in the list of genes (959, 920, 997, 797 in OFF-Crop up-regulated, DEF up-regulated, OFF-Crop down-regulated, DEF down-regulated, respectively) per GO-annotated genes (11445) in the reference list (14400 genes).

Table S5: GO[1] categorization of genes of cluster 4 of DEF buds.

General process	GO term	Description	Query item	Ref item	p-value	FDR	Enrichment fold[2]
**Light stimulus and photosynthesis**	GO:0009644	response to high light intensity	10	70	4.90E-06	0.0016	6.29
	GO:0009642	response to light intensity	11	88	6.40E-06	0.0016	5.50
	GO:0009416	response to light stimulus	22	380	0.00011	0.011	2.55
	GO:0009314	response to radiation	22	404	0.00026	0.024	2.40
**photosynthesis**	GO:0015979	photosynthesis	12	128	4.70E-05	0.0076	4.13
**Respiratory burst**	GO:0002679	respiratory burst during defense response	5	19	5.70E-05	0.0082	11.58
	GO:0045730	respiratory burst	5	20	7.40E-05	0.0096	11.00
**Abiotic stimulus**	GO:0009612	response to mechanical stimulus	5	21	9.60E-05	0.01	10.48
	GO:0009266	response to temperature stimulus	23	285	4.00E-07	0.00017	3.55
	GO:0009408	response to heat	11	99	2.00E-05	0.0043	4.89
	GO:0009628	response to abiotic stimulus	48	898	3.80E-07	0.00017	2.35
**Various**	GO:0001510	RNA methylation	8	62	8.90E-05	0.01	5.68
	GO:0006457	protein folding	14	160	2.50E-05	0.0047	3.85
	GO:0006412	translation	30	360	4.50E-09	5.90E-	3.67
	GO:0006091	generation of precursor metabolites and energy	16	252	0.00032	0.027	2.79

[1]Gene ontology
[2]Enrichmnet fold was calculated based on GO-annotated genes (260) in the list of genes (351) per GO-annotated genes (11445) in the reference list (14400 genes).

Table S6: GO[1] categorization of up-regulated genes of OFF-Crop buds.

General process	GO term	Description	Query item	Ref item	p-value	FDR	Enrichment fold[2]
**Light stimulus**	GO:0010218	response to far red light	6	25	1.50E-06	0.0013	16.54
	GO:0010114	response to red light	7	47	5.90E-06	0.0016	10.26
	GO:0009639	response to red or far red light	9	140	0.00027	0.035	4.43
**photosynthesis**	GO:0010207	photosystem II assembly	5	36	0.00018	0.027	9.57
**carbohydrate stimulus**	GO:0009744	response to sucrose stimulus	7	47	5.90E-06	0.0016	10.26
	GO:0034285	response to disaccharide stimulus	7	48	6.80E-06	0.0016	10.05
	GO:0009743	response to carbohydrate stimulus	11	178	8.70E-05	0.016	4.26

[1]Gene ontology
[2]Enrichmnet fold was calculated based on GO-annotated genes (166) in the list of genes (215) per GO-annotated genes (11445) in the reference list (14400 genes).

Table S7: GO[1] categorization of down-regulated genes of OFF-Crop buds.

General process	GO term	Description	Query item	Ref item	p-value	FDR	Enrichment fold[2]
**Response to jasmonic acid**	GO:0009867	jasmonic acid mediated signaling pathway	6	43	0.00079	0.041	5.46
	GO:0071395	cellular response to jasmonic acid stimulus	6	43	0.00079	0.041	5.46
	GO:0009753	response to jasmonic acid stimulus	14	111	1.30E-06	0.00052	4.94
**Response to ethylene**	GO:0009873	ethylene mediated signaling pathway	6	37	0.00034	0.027	6.35
	GO:0009723	response to ethylene stimulus	11	97	4.60E-05	0.0066	4.44
	GO:0071369	cellular response to ethylene stimulus	6	42	0.00069	0.04	5.59
**Amino acid metabolism**	GO:0008652	cellular amino acid biosynthetic process	11	138	0.00099	0.044	3.12
	GO:0006520	cellular amino acid metabolic process	20	270	3.60E-05	0.0058	2.90
	GO:0042398	cellular amino acid derivative biosynthetic process	15	208	0.0004	0.031	2.82
	GO:0006575	cellular amino acid derivative metabolic process	19	285	0.00021	0.02	2.61
	GO:0006519	cellular amino acid and derivative metabolic process	32	481	2.60E-06	0.00084	2.60
**Amine metabolism**	GO:0044106	cellular amine metabolic process	22	287	9.30E-06	0.0025	3.00
	GO:0009308	amine metabolic process	23	364	0.00012	0.013	2.47
**Abiotic stress**	GO:0009611	response to wounding	13	128	3.20E-05	0.0057	3.97
	GO:0009651	response to salt stress	18	292	0.00076	0.041	2.41
	GO:0006970	response to osmotic stress	19	321	0.0009	0.042	2.31
**Response to metal ions**	GO:0046686	response to cadmium ion	18	290	0.0007	0.04	2.43
	GO:0010038	response to metal ion	21	354	0.0005	0.035	2.32
**Endogenous stimulus**	GO:0071495	cellular response to endogenous stimulus	17	226	0.00011	0.013	2.94
	GO:0009719	response to endogenous stimulus	36	508	1.60E-07	0.00013	2.77
**Various**	GO:0042221	response to chemical stimulus	72	1345	7.70E-08	0.00012	2.09
	GO:0009605	response to external stimulus	20	347	0.00093	0.042	2.25
	GO:0031407	oxylipin metabolic process	7	48	0.00022	0.02	5.71
	GO:0009737	response to abscisic acid stimulus	15	225	0.0009	0.042	2.61
	GO:0019438	aromatic compound biosynthetic process	16	246	0.00081	0.041	2.54
	GO:0009725	response to hormone stimulus	29	466	2.40E-05	0.0048	2.43
	GO:0010033	response to organic substance	45	767	1.20E-06	0.00052	2.29
	GO:0044283	small molecule biosynthetic process	34	584	2.10E-05	0.0048	2.28
	GO:0019748	secondary metabolic process	27	467	0.00014	0.015	2.26
	GO:0034641	cellular nitrogen compound metabolic process	26	453	0.00021	0.02	2.24
	GO:0010035	response to inorganic substance	26	462	0.00028	0.023	2.20
	GO:0043436	oxoacid metabolic process	32	642	0.00053	0.035	1.95
	GO:0019752	carboxylic acid metabolic process	32	642	0.00053	0.035	1.95
	GO:0006082	organic acid metabolic process	32	643	0.00054	0.035	1.94
	GO:0042180	cellular ketone metabolic process	32	650	0.00065	0.04	1.92
	GO:0055114	oxidation reduction	61	1344	6.10E-05	0.0082	1.77

[1]Gene ontology
[2]Enrichmnet fold was calculated based on GO-annotated genes (292) in the list of genes (351) per GO-annotated genes (11445) in the reference list (14400 genes).

Table S8: GO[1] categorization of genes of cluster 3 of DEF buds.

General process	GO term	Description	Query item	Ref item	p-value	FDR	Enrichment fold[2]
root/root hair development	GO:0048765	root hair cell differentiation	5	79	0.00048	0.026	8.05
	GO:0048764	trichoblast maturation	5	79	0.00048	0.026	8.05
	GO:0010054	trichoblast differentiation	5	79	0.00048	0.026	8.05
	GO:0010053	root epidermal cell differentiation	5	83	0.0006	0.026	7.66
	GO:0010015	root morphogenesis	5	100	0.0014	0.038	6.36
	GO:0022622	root system development	7	175	0.00064	0.026	5.08
	GO:0048364	root development	7	175	0.00064	0.026	5.08
Floral development	GO:0048437	floral organ development	8	142	2.60E-	0.018	7.16
	GO:0048438	floral whorl development	6	114	0.00036	0.026	6.69
	GO:0009908	flower development	8	273	0.002	0.046	3.72
	GO:0048608	reproductive structure development	12	481	0.00081	0.028	3.17
	GO:0000003	reproduction	15	731	0.0015	0.039	2.61
Shoot development	GO:0010016	shoot morphogenesis	6	143	0.0012	0.038	5.33
	GO:0048367	shoot development	7	197	0.0013	0.038	4.52
	GO:0022621	shoot system development	7	199	0.0013	0.038	4.47
nucleotide metabolism	GO:0009166	nucleotide catabolic process	7	172	0.00058	0.026	5.17
	GO:0009207	purine ribonucleoside triphosphate catabolic process	7	172	0.00058	0.026	5.17
	GO:0009203	ribonucleoside triphosphate catabolic process	7	172	0.00058	0.026	5.17
	GO:0009143	nucleoside triphosphate catabolic process	7	172	0.00058	0.026	5.17
	GO:0009146	purine nucleoside triphosphate catabolic process	7	172	0.00058	0.026	5.17
	GO:0006195	purine nucleotide catabolic process	7	172	0.00058	0.026	5.17
	GO:0009154	purine ribonucleotide catabolic process	7	172	0.00058	0.026	5.17
	GO:0009261	ribonucleotide catabolic process	7	172	0.00058	0.026	5.17
	GO:0009199	ribonucleoside triphosphate metabolic process	7	217	0.0022	0.046	4.10
	GO:0009205	purine ribonucleoside triphosphate metabolic process	7	217	0.0022	0.046	4.10
	GO:0009144	purine nucleoside triphosphate metabolic process	7	217	0.0022	0.046	4.10
	GO:0009141	nucleoside triphosphate metabolic process	7	221	0.0024	0.05	4.03
	GO:0009259	ribonucleotide metabolic process	8	261	0.0015	0.04	3.90
Chromatin organization	GO:0016569	covalent chromatin modification	7	151	0.00027	0.026	5.89
	GO:0016568	chromatin modification	7	168	0.00051	0.026	5.30
	GO:0016570	histone modification	6	144	0.0012	0.038	5.30
	GO:0006325	chromatin organization	7	209	0.0018	0.045	4.26
	GO:0051276	chromosome organization	8	270	0.0019	0.046	3.77
morphogenesis and development	GO:0048646	anatomical structure formation involved in	5	87	0.00074	0.027	7.31
	GO:0048569	post-embryonic organ development	8	145	3.00E-	0.018	7.01
	GO:0021700	developmental maturation	5	93	0.001	0.033	6.83
	GO:0009887	organ morphogenesis	7	147	0.00023	0.026	6.05
	GO:0009793	embryonic development ending in seed dormancy	6	141	0.0011	0.036	5.41
	GO:0009888	tissue development	8	276	0.0022	0.046	3.68
	GO:0048513	organ development	15	563	0.00011	0.026	3.39
	GO:0048731	system development	15	566	0.00011	0.026	3.37
	GO:0009653	anatomical structure morphogenesis	11	472	0.0021	0.046	2.96
	GO:0009791	post-embryonic development	14	603	0.00066	0.026	2.95
Heterocyte	GO:0046700	heterocycle catabolic process	7	216	0.0021	0.046	4.12
	GO:0046483	heterocycle metabolic process	14	548	0.00026	0.026	3.25
ATP metabolism	GO:0006200	ATP catabolic process	6	114	0.00036	0.026	6.69
	GO:0046034	ATP metabolic process	6	156	0.0018	0.046	4.89
Various	GO:0048469	cell maturation	5	80	0.00051	0.026	7.94
	GO:0009733	response to auxin stimulus	6	131	0.00076	0.027	5.82
	GO:0009314	response to radiation	13	404	4.60E-	0.018	4.09
	GO:0009266	response to temperature stimulus	9	285	0.00067	0.026	4.01
	GO:0009416	response to light stimulus	11	380	0.00039	0.026	3.68
	GO:0071310	cellular response to organic substance	9	314	0.0013	0.038	3.64
	GO:0006812	cation transport	9	333	0.0019	0.046	3.44
	GO:0051716	cellular response to stimulus	16	750	0.00073	0.027	2.71
	GO:0009628	response to abiotic stimulus	19	898	0.0003	0.026	2.69
	GO:0042221	response to chemical stimulus	23	1345	0.0014	0.038	2.17

[1]Gene ontology

[2]Enrichmnet fold was calculated based on GO-annotated genes (90) in the list of genes (104) per GO-annotated genes (11445) in the reference list (14400 genes).

Table S9: GO[1] categorization of genes of cluster 2 of ON-Crop buds.

General process	GO term	Description	Query item	Ref item	p-value	FDR	Enrichment fold[2]
**Lipid transport**	GO:0006869	lipid transport	10	66	6.10E-09	4.00E-06	13.33
	GO:0010876	lipid localization	10	84	6.50E-08	2.20E-05	10.48
**Various**	GO:0009409	response to cold	9	172	0.00022	0.036	4.60
	GO:0006412	translation	15	360	3.60E-05	0.0079	3.67

[1]Gene ontology
[2]Enrichmnet fold was calculated based on GO-annotated genes (130) in the list of genes (191) per GO-annotated genes (11445) in the reference list (14400 genes).

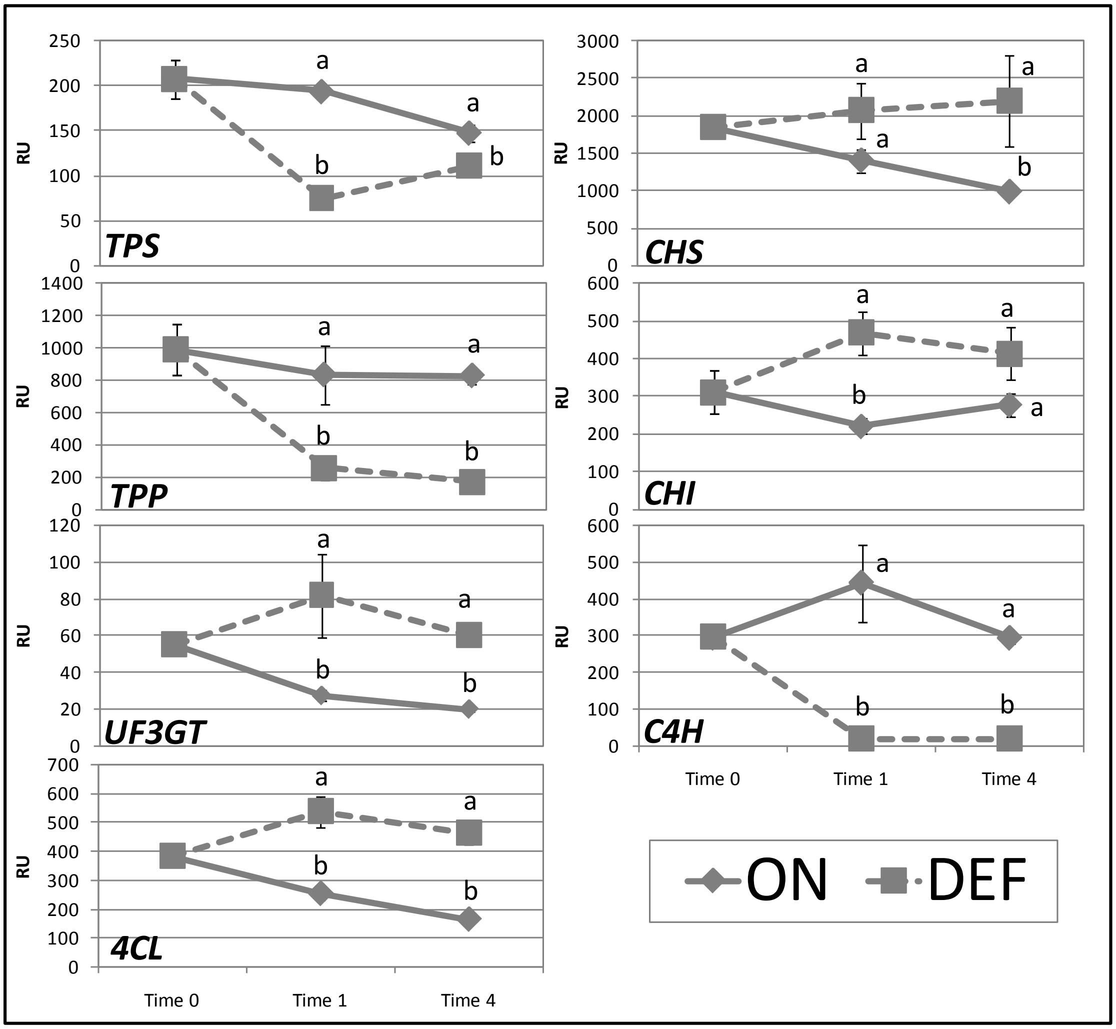

Figure S1. De-fruiting alters the expression of trehalose and flavonoid metabolizing genes. The mRNA levels of trehalose phosphate phosphatase (TPP) , trehalose phosphate synthase (TPS), chalcone synthase (*CHS*), cinnamate 4-hydroxylase (*C4H*), 4-coumarate:coenzyme A ligase (*4CL*), chalconeisomerase (CHI), and UDP-glucose:flavonoid-3-O-glucosyltransferase (*UF3GT*) were measured in buds of ON-Crop (ON) and de-fruited trees at the indicated times following fruit removal by nCounter analyses. The numbers are mean values of three independent biological replicates ± SE .

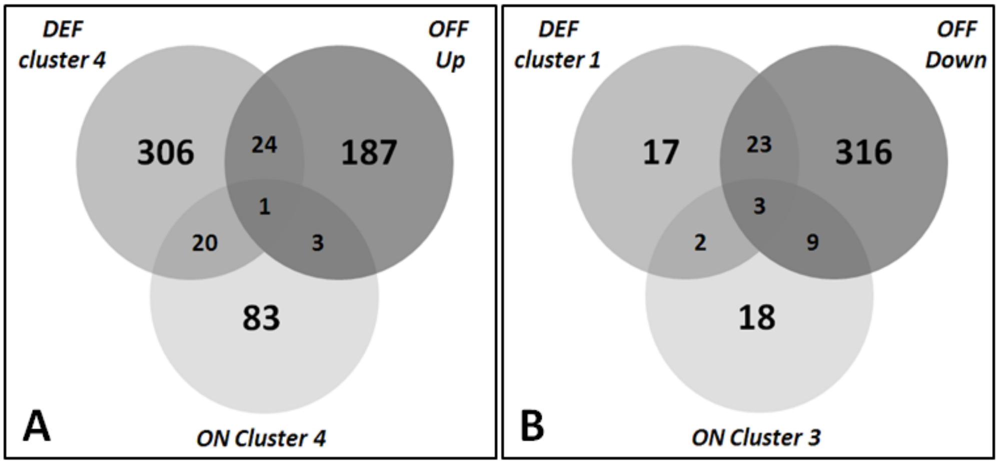

Figure S2. Venn diagrams of developmentally-regulated genes. Specific genes of the indicated clusters are listed in Supp. File 2.

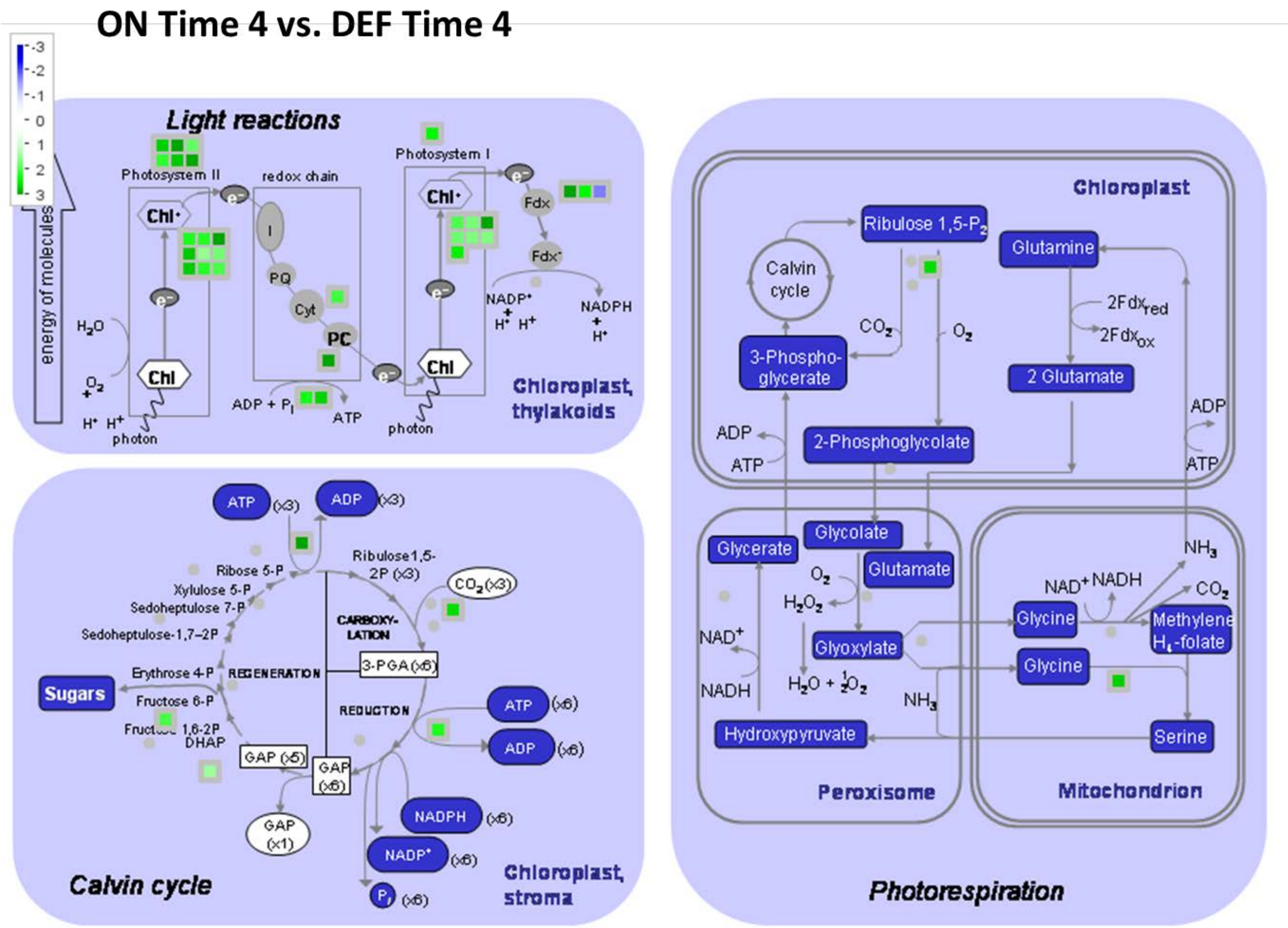

Figure S3. Photosynthetic genes are induced following de-fruiting in the bud. Differentially expressed genes were analyzed by MapMan. Green squares represent genes reduced in buds of ON-Crop (ON) trees relative to buds of de-fruited (DEF) trees. A description of the specific genes and their fold change is provided in Supp. File 2 (under Fig. 5A).

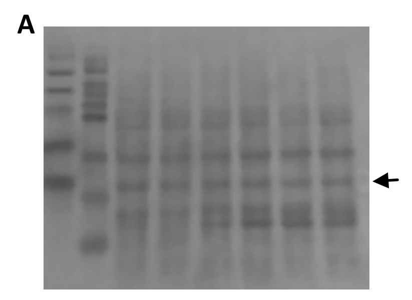

B

	ON 1	ON 2	DEF 1	DEF 2	OFF 1	OFF 2
**RbcL**	1.27	0.83	0.96	0.86	0.87	1.17
**RbcS**	0.67	0.52	0.95	0.92	1.31	1.95
**Fd**	0.89	0.79	0.90	0.99	1.22	1.36
**LHCII**	0.72	0.54	0.85	1.11	1.08	0.99
**PsbA**	0.67	0.74	0.94	1.22	1.37	1.29

Figure S4. Quantification of protein blot results (Fig. 5C). Following electro-transfer, the protein blot was dyed with Ponceau S (A). Quantification of the protein signals following chemoiluminescence reaction (B) was carried on using the arrow-marked bend (A) as a standard.

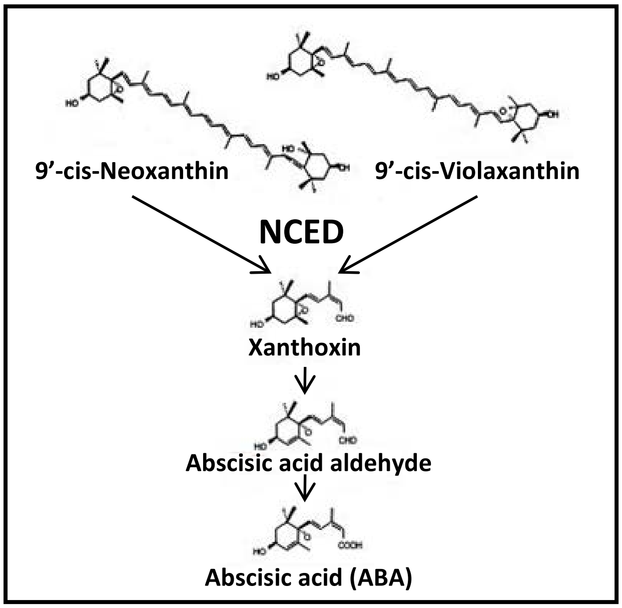

Fig.S5. Schematic representation of the cleavage of 9-cis xanthophylls to xanthoxin by 9-cis-epoxycarotenoid dioxygenase (NCED), a key regulated step in the biosynthesis of ABA in plants

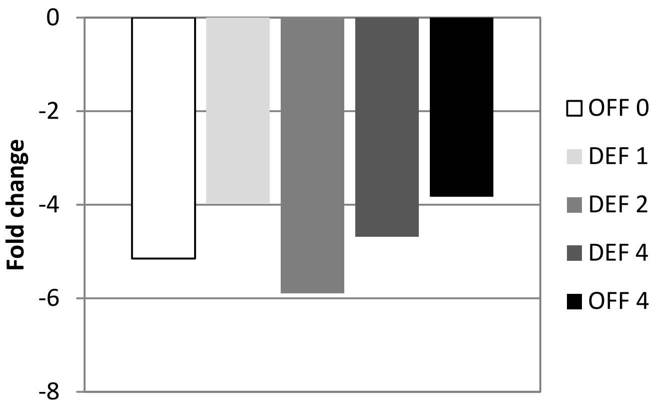

Figure S6. Changes in the expression of *PYR1-like* gene. Fold change in transcript levels of *Cs7g30500.1*, showing 84% homology (73% identity) to the *Arabidopsis* gene At2g38310 encoding a PYR protein, an ABA receptor component.

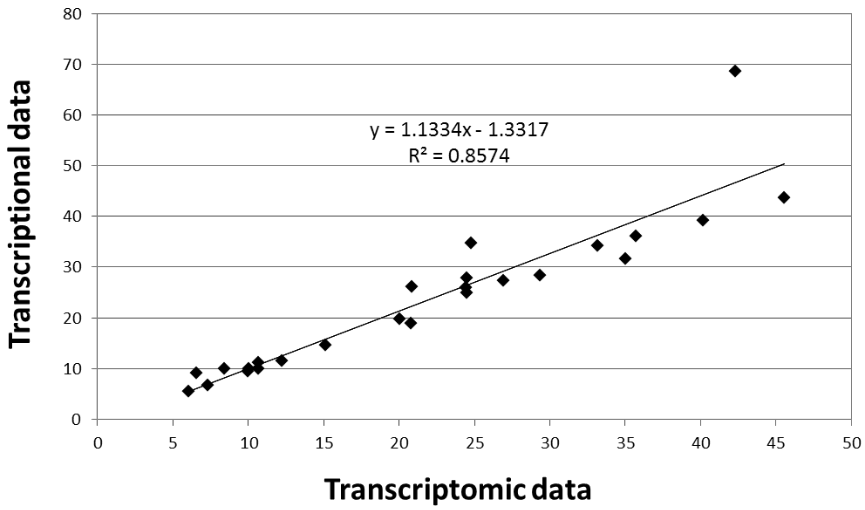

Figure S7. Linear regration between transcriptomic and transcriptional (qPCR) data of Ca-related and NPH3-like genes.

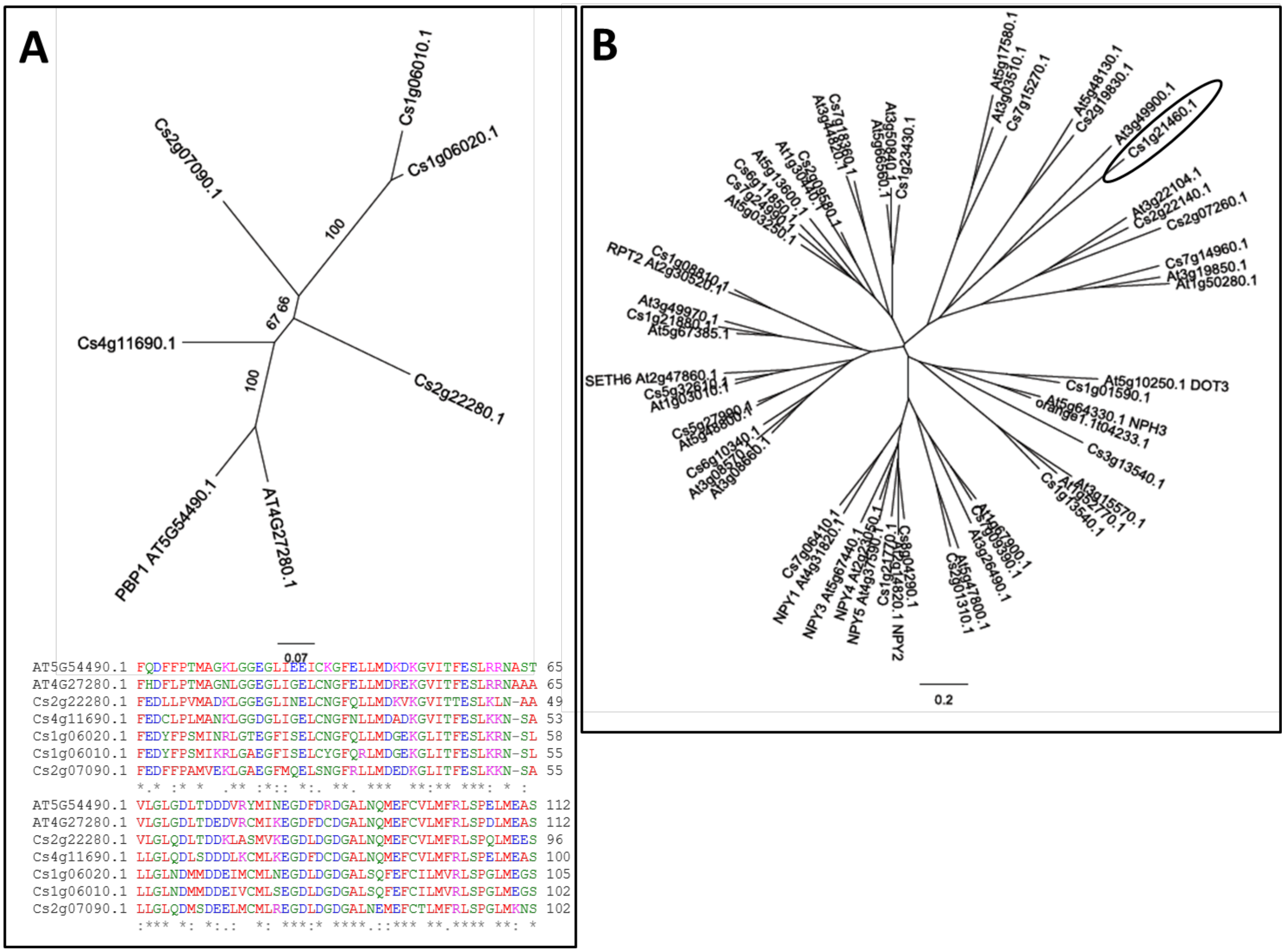

Fig.S8. Genes encoding PINOID (PID)-binding proteins 1 (PBP1) and Non-Phototropic Hypocotyl 3-like (NPH3) show homology in *Arabidopsis* and *Citrus*. Phylogenic analyses of the *PBP1-like* (A) and NPH3-*like* (B) gene families from *Arabidopsis* and *Citrus*. The circle in B marks the citrus gene analyzed in Fig. 8. Alignments were performed by MUSCLE program using default parameters (http://www.ebi.ac.uk/Tools/msa/muscle/ Edgar 2004). Phylogenetic trees were constructed based on maximum likelihood (ML) framework using the phyml software (Guindon & Gascuel, 2003) by the JTT matrix-based model (Jones *et al*. 1992). Bootstrap consensus tree was inferred from 100 replicates (Felsenstein *et al*. 1985) and the percentage of replicate trees in which the associated clade clustered together in the bootstrap test is designated next to the branches (Felsenstein *et al*. 1985). The trees were graphically designed with the use of FigTree version 1.4 (http://tree.bio.ed.ac.uk/software/figtree/).
